# Co-Treatment With Verapamil and Curcumin Attenuates the Behavioral Alterations Observed in Williams–Beuren Syndrome Mice by Regulation of MAPK Pathway and Microglia Overexpression

**DOI:** 10.3389/fphar.2021.670785

**Published:** 2021-08-03

**Authors:** Paula Ortiz-Romero, Alejandro González-Simón, Gustavo Egea, Luis A. Pérez-Jurado, Victoria Campuzano

**Affiliations:** ^1^ Departament de Biomedicina, Facultat de Medicina i Ciències de la Salut, Universitat de Barcelona, Barcelona, Spain; ^2^ Institut d’Investigacions Biomèdiques August Pi I Sunyer, IDIBAPS-UB, Barcelona, Spain; ^3^ Unitat de Genètica, Departament de Ciències Experimentals i de la Salut, Universitat Pompeu Fabra, Barcelona, Spain; ^4^ Centro de Investigación Biomédica en Red de Enfermedades Raras (CIBERER), ISCIII, Barcelona, Spain; ^5^ Servei de Genètica, Hospital del Mar, Institut Hospital del Mar d’Investigacions Mèdiques (IMIM), Barcelona, Spain

**Keywords:** Williams–Beuren syndrome, curcumin, verapamil, mice, activated glia, behavior

## Abstract

Williams–Beuren syndrome (WBS) is a rare neurodevelopmental disorder characterized by a distinctive cognitive phenotype for which there are currently no effective treatments. We investigated the progression of behavioral deficits present in WBS complete deletion (CD) mice, after chronic treatment with curcumin, verapamil, and a combination of both. These compounds have been proven to have beneficial effects over different cognitive aspects of various murine models and, thus, may have neuroprotective effects in WBS. Treatment was administered orally dissolved in drinking water. A set of behavioral tests demonstrated the efficiency of combinatorial treatment. Some histological and molecular analyses were performed to analyze the effects of treatment and its underlying mechanism. CD mice showed an increased density of activated microglia in the motor cortex and CA1 hippocampal region, which was prevented by co-treatment. Behavioral improvement correlated with the molecular recovery of several affected pathways regarding MAPK signaling, in tight relation to the control of synaptic transmission, and inflammation. Therefore, the results show that co-treatment prevented behavioral deficits by recovering altered gene expression in the cortex of CD mice and reducing activated microglia. These findings unravel the mechanisms underlying the beneficial effects of this novel treatment on behavioral deficits observed in CD mice and suggest that the combination of curcumin and verapamil could be a potential candidate to treat the cognitive impairments in WBS patients.

## Introduction

Williams–Beuren syndrome (WBS, OMIM 194050) is a rare neurodevelopmental disorder with an estimated prevalence of 1 in 7,500–20,000 newborns that is caused by the heterozygous deletion of 26–28 contiguous genes (1.55–1.83 Mb) on chromosome 7q11.23 (Strømme et al., 2002; Bayés et al., 2003). Together with some cardiovascular features, WBS individuals present mild to moderate intellectual disability, with a mean intelligence quotient (IQ) that ranges between 50 and 60 (Martens et al., 2008). They present a unique cognitive phenotype that includes severe deficits in visuospatial construction and increased sociability, together with preserved linguistic abilities (Bellugi et al., 2000; Doyle et al., 2004). Given the complexity of the cognitive profile of WBS patients, they often require assistance of a mental health professional. However, pharmacological intervention is usually inaccurate and only targeted to treat anxiety (Green et al., 2012; Martens et al., 2012). For this reason, it is interesting to study any potential treatment that might improve the cognitive impairments observed in WBS.

The complete deletion (CD) mouse model presents a cognitive phenotype very similar to that seen in humans with WBS (Segura-Puimedon et al., 2014) and has been used to define possible therapeutic interventions (Borralleras et al., 2015; Ortiz-Romero et al., 2018). The cognitive characterization of this animal model has shown impairments in motor coordination, anxiety-like behavior, and hypersociability (Segura-Puimedon et al., 2014). Moreover, histological analyses of the brain have revealed disorganization in certain brain areas as well as an abnormal morphology of dendritic spines (Borralleras et al., 2015; Ortiz-Romero et al., 2018).

In the last years, the study of the therapeutic effects of natural phenols has gained attention. Accumulated evidence has described that curcumin, the major constituent of turmeric (*Curcuma longa*), exerts a variety of pharmacological effects due to its antioxidant, anti-inflammatory, and neuroprotective properties (Hay et al., 2019). Recent studies have reported positive effects of curcumin over different cognitive aspects such as anxiety-like behaviors, memory deficits, and motor impairments of different murine models (Spinelli et al., 2015; Aubry et al., 2019; Sanei and Saberi-Demneh, 2019). Many studies have described that its effects on the behavioral phenotype of mice models are mediated by upregulation of BDNF (brain-derived neurotrophic factor) expression (Zhang et al., 2012; Franco-Robles et al., 2014; Nam et al., 2014). BDNF has been described as a crucial molecule for neural development and plasticity processes (von Bohlen und Halbach, 2018), and its mechanism of action is highly dependent on a proper maintenance of intracellular ionic homeostasis (Li et al., 1999; Numakawa et al., 2001). Moreover, it has also been described to prevent neuroinflammation by modulating pathways related to NRF2 and MAPK signaling (Jin et al., 2018).

Verapamil is a widely used medication, and its mechanism of action involves mainly the blocking of voltage-dependent calcium channels (Elliott and Ram, 2011), but it has also been proven to directly bind and block voltage-gated potassium channels (Ko et al., 2010) and to inhibit drug efflux pump proteins like P-glycoprotein (Bellamy, 1996). Although it has been mainly studied for cardiovascular applications, it has also been associated with positive effects on anxiety and memory processing in murine models (Quartermain and Garcia DeSoria, 2001; Biala and Kruk, 2008).

Given the properties of both compounds, we decided to explore the effects of each compound and a combinatorial treatment on the behavioral phenotype of CD mice. The results show that only the combined treatment with curcumin and verapamil improved the deficits. This improvement can be correlated with the normalization of the MAPK and inflammasome signaling pathways and with the concomitant reduction of activated microglia.

## Materials and Methods

### Ethics Statement

The local Committee of Ethical Animal Experimentation (CEEA-PRBB Protocol Number: VCU-17-0021; University of Barcelona Protocol Number: Campuzano-153.19) and Generalitat de Catalunya (Protocol Number: DAAM-9494) approved all animal procedures in accordance with the guidelines of the European Commission Directive 86/609/EEC. The PRBB has Animal Welfare Assurance (#A5388-01, Institutional Animal Care and Use Committee approval date 05/08/2009), granted by the Office of Laboratory Animal Welfare (OLAW) of the US National Institutes of Health.

### Animals’ Maintenance

CD mice, a WBS murine model that carries a 1.3 Mb heterozygous deletion harboring from *Gtf2i* to *Fkbp6*, were obtained as previously described (Segura-Puimedon et al., 2014). CD mice were crossed with Thy1-YFP transgenic mice (B6.Cg-Tg(Thy1-YFPH)2Jrs/J, The Jackson Laboratory) (Feng et al., 2000) to label pyramidal neurons. All mice were maintained on 97% C57BL/6J background. Genomic DNA was extracted from mouse ear punch to perform genotyping using MLPA/PCR and appropriate primers, as previously described (Borralleras et al., 2015). Animals were housed under standard conditions in a 12 h dark/light cycle with access to food and water/treatment *ad libitum*. We used four groups of treatment (vehicle (VEH), verapamil (VER), curcumin (CUR), and combined (VERCUR)) per genotype (WT and CD) in male mice, with n = 7–11 in all groups.

### Treatment Administration and Intake

Considering that the higher dose of VER used in human patients is 480 mg/day, we established an approximately equivalent dose of 7 mg/kg/day for our treatment (considering average mouse weight 25 g). VER was dissolved in drinking water and kept at -21°C until its use. CUR was administered at a dose of 60 mg/kg/day as previously reported (Babu et al., 2015). It was freshly prepared once per week, from a curcumin extract (Super Bio-Curcumin^®^, from Life Extension^®^, United States, total curcuminoid complex with essential oils of turmeric rhizome by HPLC (400 mg)) dissolved in drinking water with 1% DMSO.

The VEH control group consisted of WT and CD mice drinking water with 1% DMSO. Experimental groups consisted of 1) WT and CD mice treated with VER only, 2) WT and CD mice treated with CUR only, and 3) WT and CD mice treated with both compounds (VERCUR).

Respective treatments were started at 8–9 weeks old. All animals received their treatments for 5 weeks before starting the behavioral tests, and it was maintained for the whole duration of these procedures until sacrifice at 13–14 weeks old. The amount of drink per cage was quantified and normalized to the number of animals per cage (2–4) and to the time between each change (48–60 h). Consumption was not significantly different between groups (F_2.005,30.08_ = 0.4796, *p* = 0.6242) (Supplementary Figure 1A). Daily intake was not significantly different among groups (F_3,41_ = 0.9419, *p* = 0.4292). A significant effect of genotype is observed because CD mice drink less regardless of treatment (F_1,41_ = 8.402, *p* = 0.006) (Supplementary Figure 1B).

None of the treatments changed the reduced body weight presented by CD mice compared to WT mice (effect of genotype F_1,84_ = 146.4, *p* < 0.0001) (Supplementary Figure 1C).

### Behavioral Tests

To evaluate the therapeutic potential of treatments, we have chosen behavioral tests that have revealed altered phenotypes in CD mice based on previous data (Segura-Puimedon et al., 2014; Borralleras et al., 2015). These phenotypes include motor coordination deficits (rotarod test), hypersociability (the direct social interaction test for social habituation), and altered naturally occurring behaviors (marble-burying test). All the experiments were performed during the light phase of the dark/light cycle by researchers blind to the different experimental groups. There was a constant illumination of about <10 lux. All the apparatuses were cleaned after each mouse.

#### Social Interaction Test

The social interaction test was conducted in an open field as previously described (Borralleras et al., 2015). Briefly, an empty wire cup container was placed in the center of the arena, and each individual mouse was allowed to freely explore the arena for 5 min. During this time, the amount of time sniffing the empty container was measured. After this, a stimulus mouse (WT, age and sex matched to the experimental mice) was placed in the container, and again, the amount of time nose-to-nose sniffing the stimulus mouse was measured during that 5 min. The time spent exploring each situation (empty and social stimuli) during the test session was computed to calculate a preference score. A higher preference score is considered to reflect greater social interest. To avoid tiredness, different stimulus mice were used (each stimulus mouse was used no more than twice a day with an interval of at least 60 min).

#### Marble-Burying Test

The marble-burying test was conducted as previously described (Ortiz-Romero et al., 2018). Briefly, a polycarbonate rat cage was filled with 5 cm depth of bedding and lightly tamped down. A regular pattern of 20 glass marbles (five rows of four marbles) was placed on the surface of the bedding prior to each test. Animals were tested individually, and the number of buried marbles (considered buried when >2/3 of the marble was covered) was counted after 20 min.

#### Rotarod Test

The rotarod test was performed as previously described (Borralleras et al., 2015). Briefly, the test was conducted using a Rotarod LE8500 apparatus (Panlab, Harvard Apparatus). First, a period of training was performed in which animals were trained until they could stay on the rod for 120 s at the minimum speed (4 rpm). After 5 min of rest, the test was started, which consisted in measuring the latency to fall off the rod in consecutive trials of 7, 10, 14, 19, 24, 34, and 40 rpm, with a maximum of 60 s per trial and a rest of 5 min between trials.

### Histological Preparation

Animals were perfused with 1X phosphate-buffered saline (PBS) followed by 4% paraformaldehyde (PFA). Brains were removed and postfixed in 4% PFA for 24 h at 4°C and in PBS for 24 h at 4°C; afterward, they were cryoprotected in 30% sucrose for 24 h at 4°C.

#### Immunofluorescence

30 μm-thick coronal brain sections were permeabilized with 0.3% Triton-X and incubated with NH_4_Cl 50 mM for 30 min at room temperature. Blocking was performed with a solution of 2% bovine serum albumin and 3% fetal bovine serum for 2 h at room temperature. Afterward, sections were incubated overnight at 4°C with primary antibodies [rabbit polyclonal anti-IBA1 (1:1,000, Wako) and goat polyclonal anti-IL1β (1:500, R&D Systems)]. After washing, secondary antibodies [1:500 anti-rabbit Alexa Fluor 555, anti-goat Alexa Fluor 405 (Invitrogen)] were incubated for 1 h at room temperature. The sections were mounted with the Mowiol mounting medium.

For IBA1/IL1β quantification, 1024 × 1024 pixel confocal fluorescent image stacks from these brain sections were obtained with a TCS SP5 LEICA confocal microscope, using an X20 objective. We obtained pictures of the CA1 hippocampus and motor cortex. ImageJ software (https://imagej.nih.gov/ij/) was used for image quantification. N = 5–7 animals per group were analyzed, and three brain slices were analyzed per animal.

#### Morphological Evaluation of Different Brain Areas

150 μm-thick serial coronal brain sections were collected on a glass slide and directly mounted with Mowiol.

For the quantification of the number of YFP + neurons, 1360 × 1024 pixel images of the CA1 hippocampus and motor cortex were obtained with an Olympus DP71 camera attached to an Olympus BX51 microscope with an Olympus U-RFL-T source of fluorescence at 10x magnification. ImageJ software was used for quantification. N = 3–5 animals per group were analyzed.

For spine density and length analyses, 1024 × 1024 pixel confocal fluorescent image stacks from these tissue sections were obtained with a TCS SP2 LEICA confocal microscope, using an X63 (zoom x5) oil immersion objective. We obtained pictures of dendritic segments of 15–30 µm from randomly selected neurons in CA1 hippocampus and motor cortex sections. ImageJ software was used for quantification. Spine counts included all types of dendritic protrusions. N = 3–4 animals per group were analyzed, and ten dendritic segments were analyzed per animal. Spine density was calculated by relativizing the total number of spines to the length of the analyzed dendrite. Spine length was measured from the base of the spine to the end of the head of the spine.

### RNA Extraction and Gene Expression Analyses

Brains were removed, and the frontal cortex was isolated, immediately frozen, and kept at −80°C until use. RNA was extracted from the cerebral cortex using the RNeasy Mini Kit (Qiagen) following the manufacturer’s instructions, and mRNA quality was evaluated with the Bioanalyzer (Agilent).

RNA from WT and CD mice (n = 3 each) per experimental group (VEH- and VERCUR-treated) was used to prepare twelve mRNA libraries following the standard Illumina protocol. Libraries were sequenced at the Centre Nacional d’Anàlisi Genòmica (CNAG-CRG, Barcelona, Spain) facilities, using an Illumina HiSeq2000 platform to produce over 60 million paired-end reads (100 nucleotide length) per sample. Reads were mapped to the Ensembl GRCm38 *Mus musculus* genome provided by Illumina iGenomes. Mapping was performed with STAR/2.5.3a. After a correction by percentage of coefficient of variation (%CV < 20), we discriminated 13941 mapped protein coding genes to be analyzed. We used a general linear model (GLM) to assess differential expression, and correction of the *p* values was then calculated using the Wald test. The differentially expressed genes were selected based on criteria combining either an increase of 25% (FC > 1.25) or a decrease of 25% (FC < 0.75) and an adjusted *p* value of 0.05. Expression values were relativized according to the average expression of the WT animals for each gene. Results were validated by the expression analysis of the genes included in the WBS critical region (WBSCR), 19 of which are represented in the analysis (Supplementary Figure 2) and are known to present reduced expression in WBS mice models (Li et al., 2009; Segura-Puimedon et al., 2014; Kopp et al., 2019). We used the CPDB (available at http://www.consensuspathdb.org/) to investigate the functional associations of genes found to be either down- or upregulated. RNA-seq data were deposited on Gene Expression Omnibus (GEO) and made publicly available (GSE 162092).

### Protein Extraction and Western Blot

Frozen brain areas were dounce-homogenized in 30 volumes of lysis buffer (50-mmol/L Tris-HCl of pH 7.4, 150-mmol/L NaCl, 10% glycerol, 1-mmol/L EDTA, 10-μg/ml aprotinin, 1-μg/ml leupeptin, 1-μg/ml pepstatin, 1-mmol/L phenylmethylsulfonyl fluoride, 1-mmol/L sodium orthovanadate, 100-mmol/L sodium fluoride, 5-mmol/L sodium pyrophosphate, and 40-mmol/L beta-glycerolphosphate) plus 1% Triton X-100. After 10 min incubation at 4°C, the samples were centrifuged at 16,000 g for 20 min to remove insoluble debris. Protein contents in the supernatants were determined by a DC microplate assay (Bio-Rad, Madrid, Spain), following the manufacturer’s instructions.

The antibodies used for western blot were TGFβ (1:1,000, rabbit, Abcam), pAKT (1:1,000, mouse, Cell Signaling Technology), BDNF (1:1,000, rabbit, Santa Cruz Biotechnology), and actin (1:10000, rabbit, Sigma). Primary antibodies were detected with horseradish peroxidase–conjugated antibody (1:2,000, Promega) and visualized by enhanced chemiluminescence detection (Western Blotting Luminol Reagent, Santa Cruz). Digital images were acquired on the ChemiDoc XRS System (Bio-Rad) and quantified by ImageJ software. Optical density values for target proteins were normalized to those of Ponceau or actin loading control in the same sample, and they were expressed as a percentage of control group (VEH).

### Statistical Analysis

All data are presented as mean ± SEM. In all cases, we have analyzed normality and equality of variances using Shapiro–Wilk and Levene tests, respectively, with SPSS software. Parametric (two-way ANOVA followed by Bonferroni’s *post hoc* test) and non-parametric (Mann–Whitney, Kruskal–Wallis, and Friedman tests followed by Dunn’s *post hoc* test) tests were used when needed. Values were considered significant when *p* < 0.05. GraphPad Prism 9 software was used for obtaining all statistical tests and graphs. Heatmap was generated using heatmap.2 function of the *gplots* package in R.

## Results

### Only VERCUR Co-Treatment Improves Motor Coordination in CD Mice

**FIGURE 1:**
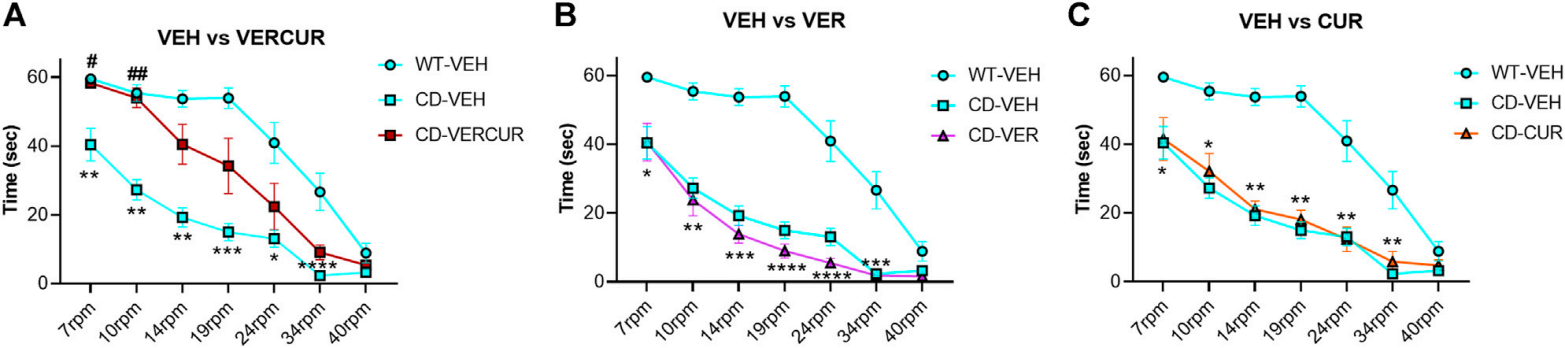
Only VERCUR co-treatment improves motor coordination in CD mice. **(A)** After combinatorial VERCUR treatment, CD animals performed the rotarod test similar to VEH-treated WT mice. **(B)** Single treatment with VER did not have any effect on the performance of treated CD animals in the rotarod test. **(C)** Single treatment with CUR did not have any effect on the performance of treated CD animals in the rotarod test. Data are presented as the mean ± SEM of n = 9–11 mice. *p* values are shown with asterisks (compared to VEH-treated WT) and hashes (compared to VEH-treated CD) indicating values that are significantly different in individual group comparisons (Friedman test, followed by the Kruskal–Wallis test with Dunn’s *post hoc* test). ^*,#^ *p* < 0.05; ^**,##^ *p* < 0.01; ^***^ *p* < 0.001; ^****^ *p* < 0.0001.

Motor coordination was evaluated after 5-weeks of treatment in the rotarod test in WT and CD mice receiving VEH, VER, CUR, or VERCUR. The Friedman test revealed significant differences (χ^2^ (7) = 42.24, *p* < 0.0001) among groups. As expected from previous results (Segura-Puimedon et al., 2014), we observed significant motor coordination deficits in the VEH-treated CD animals in comparison with WT animals from 7 to 34 rpm (Figure 1). However, only the VERCUR co-treated group revealed significant effects of treatment showing a behavior similar to that of VEH-treated WT mice and reaching significant differences with respect to VEH-treated CD mice at 7 and 10 rpm (*p* = 0.0326 and *p* = 0.0032, respectively) (Figure 1A). The results show neither single VER nor single CUR treatments had any significant effect on the performance of treated animals in this test (Figures 1B,C). None of the treatments had any effect on the performance of WT mice in this test (Supplementary Figure 3). Detailed statistical analysis is shown in Supplementary Table 1.

### Combinatorial Treatment Prevents Hypersociability of CD Mice

**FIGURE 2:**
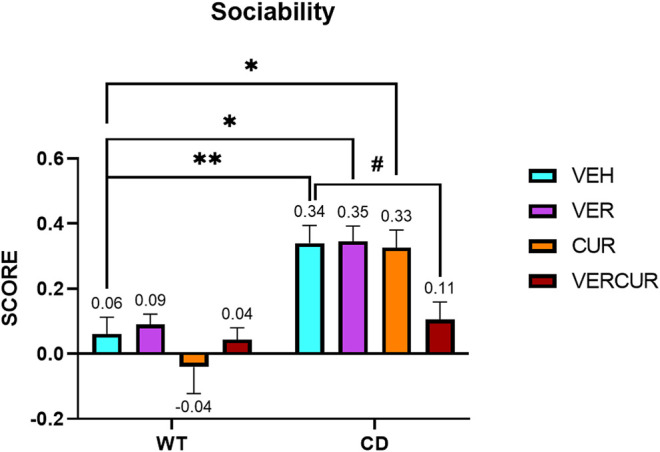
Combinatorial treatment prevents hypersociability of CD mice. In a direct social test, VEH-treated CD animals presented an increased interest toward a stimulus mouse in comparison with the VEH-treated WT mice (*p* = 0.0064), while VERCUR-treated CD animals showed a preference score similar to that of VEH-treated WT mice (*p* > 0.9999). Single VER or CUR treatments did not have any effect on the performance of both WT and CD mice in this test. Data are presented as the mean ± SEM of n = 9–11 mice. *p* values are shown with asterisks (effect of genotype) and hashes (effect of treatment) indicating values that are significantly different in individual group comparisons (Bonferroni’s *post hoc* test). ^**^ *p* < 0.01; ^*,#^ *p* < 0.05.

Social behavior was determined using the direct social interaction test as previously described (Segura-Puimedon et al., 2014). As expected, replicating previous results, two-way ANOVA revealed significant interaction (genotype/treatment) (F_3,69_ = 2.876; *p* = 0.0423). Subsequent analyses corrected for the multiple-comparisons test (Bonferroni) revealed that the hypersocial behavior was prevented by VERCUR co-treatment (*p >* 0.999) (Figure 2), but not by single VER (*p* = 0.0133) or CUR (*p* = 0.0211) treatment in which treated animals replicated the behavior of VEH-treated mice (*p* = 0.0064) (Figure 2). None of the treatments affects the WT behavior. Detailed statistical analysis is shown in Supplementary Table 2. No differences were observed, neither in genotype (F_1,69_ = 1.235; *p* = 0.2704) nor in treatment (F_3,69_ = 1.166; *p* = 0.3291) in the activity of the animals during the social interaction test measured as the total time that the animals spent exploring (Supplementary Figure 4 and Supplementary Table 2).

### MBT of CD Animals Is Not Improved by Any Treatment

**FIGURE 3:**
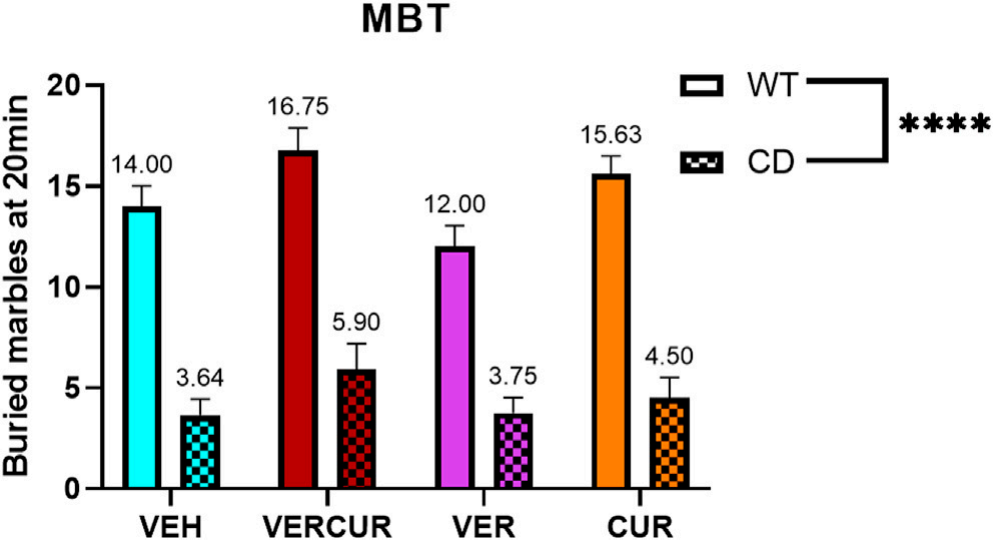
MBT of CD animals is not improved by any treatment. The number of buried marbles at the end point of the marble-burying test was significantly lower in CD animals compared to WT animals (effect of genotype: F_1,70_ = 197.9; *p* < 0.0001), and this effect was not prevented by any of the treatments (effect of treatment: F_3,70_ = 4.240; *p* = 0.0082). Data are presented as the mean ± SEM of n = 7–11 mice. *p* values are shown with asterisks indicating values that are significantly different in two-way ANOVA. ^****^ *p* < 0.0001.

The evaluation of naturally occurring behavior was performed by counting the number of marbles buried in the marble-burying test (MBT) after 5 weeks of treatment. Two-way ANOVA (genotype, treatment) revealed significant effects of genotype (F_1,70_ = 197.9.2; *p* < 0.0001) as CD animals performed this test burying significantly less marbles than the WT animals, as has been previously described (Segura-Puimedon et al., 2014). This effect was not prevented by any of the treatments (interaction F_3,70_ = 0.7893; *p* = 0.5039) (Figure 3). Detailed statistical analysis is shown in Supplementary Table 3.

### Neuroanatomical Features of CD Mice Do Not Change After VERCUR Co-Treatment

The neurocognitive profile of WBS patients has been related with abnormalities in the structure of the hippocampus and the cerebral cortex (Reiss et al., 2000). Previous studies showed that CD animals present significant alterations in different cortical and hippocampal regions (Segura-Puimedon et al., 2014; Borralleras et al., 2015; Ortiz-Romero et al., 2018; Dasilva et al., 2020). Substantial structural reorganization of various areas of the adult mouse brain (including the hippocampus and motor cortex) has been described after a relatively short period of motor skills training using the rotarod (Scholz et al., 2015). The aforementioned positive effects of VERCUR co-treatment led us to analyze the characteristics of the motor cortex (MC) and the CA1 hippocampal region (HPC) of co-treated CD mice to evaluate if this was correlated with any changes at a neuroanatomical level.

**FIGURE 4:**
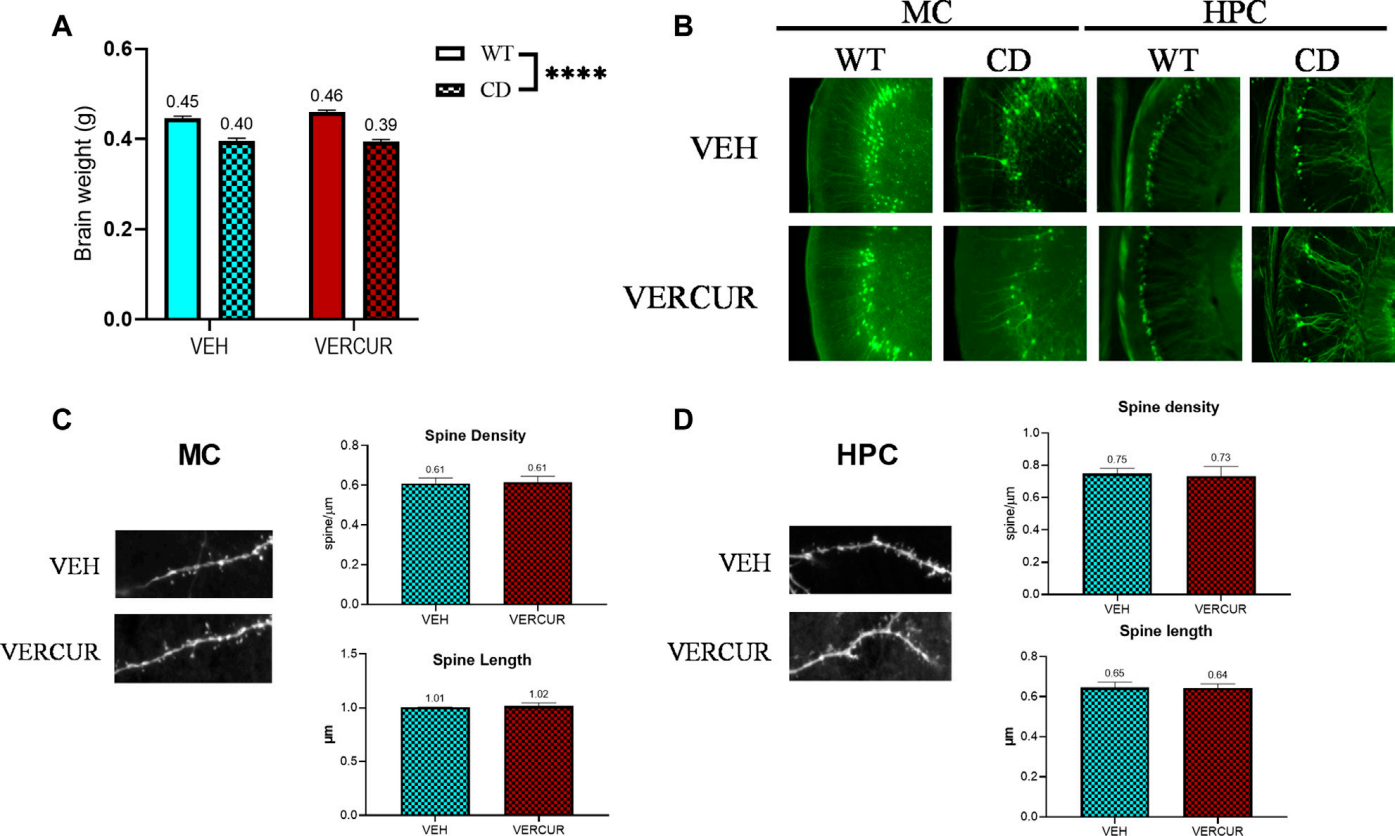
Neuroanatomical features of CD mice do not change after VERCUR co-treatment. **(A)** Brain weight of VERCUR-treated CD animals was significantly reduced when compared to that of WT mice. A two-way ANOVA indicated a significant effect of genotype (F_1,67_ = 171.2, *p* < 0.0001) but no effect of treatment (F_3,67_ = 0.2753, *p* = 0.8431). Data are presented as the mean ± SEM of n = 13–15 mice. *p* values are shown with asterisks indicating values that are significantly different in the two-way ANOVA test. ^****^ *p* < 0.0001. **(B)** Representative images of the motor cortex and hippocampus of VEH- and VERCUR-treated WT and CD animals. **(C, D)** Representative images and quantification of spine density and spine length in the motor cortex **(C)** and hippocampus **(D)** of VEH- and VERCUR-treated CD animals. Data are presented as the mean ± SEM of n = 3-4 mice. Comparison with the Mann–Whitney test showed no significant differences.

We observed that none of the treatments had any effect on the recovery of a normal brain weight with a significant effect of genotype (F_1,67_ = 171.2, *p* < 0.0001) but no effect of treatment (F_3,67_ = 0.2753, *p* = 0.8431) (Figure 4A, Supplementary Figure 5A). In addition, CD animals presented a significant reduction of the number of YFP + neurons in both the MC (effect of genotype: F_1,25_ = 168.5, *p* < 0.0001) and the HPC (effect of genotype: F_1,25_ = 218.8, *p* < 0.0001). None of the treatments had any effect on the recovery of the number of YFP + neurons, neither in the MC nor in the HPC (Figure 4B; Supplementary Figures 5B,C). Moreover, the analysis of spine density and length of VERCUR- versus VEH-treated CD mice revealed no differences neither in the MC nor in the HPC (Figures 4C,D). Detailed statistical analysis is shown in Supplementary Table 4.

### The Increased Microglia Activation in Motor Cortex and Hippocampus Presented by CD Mice Is Prevented by VERCUR Co-Treatment

**FIGURE 5:**
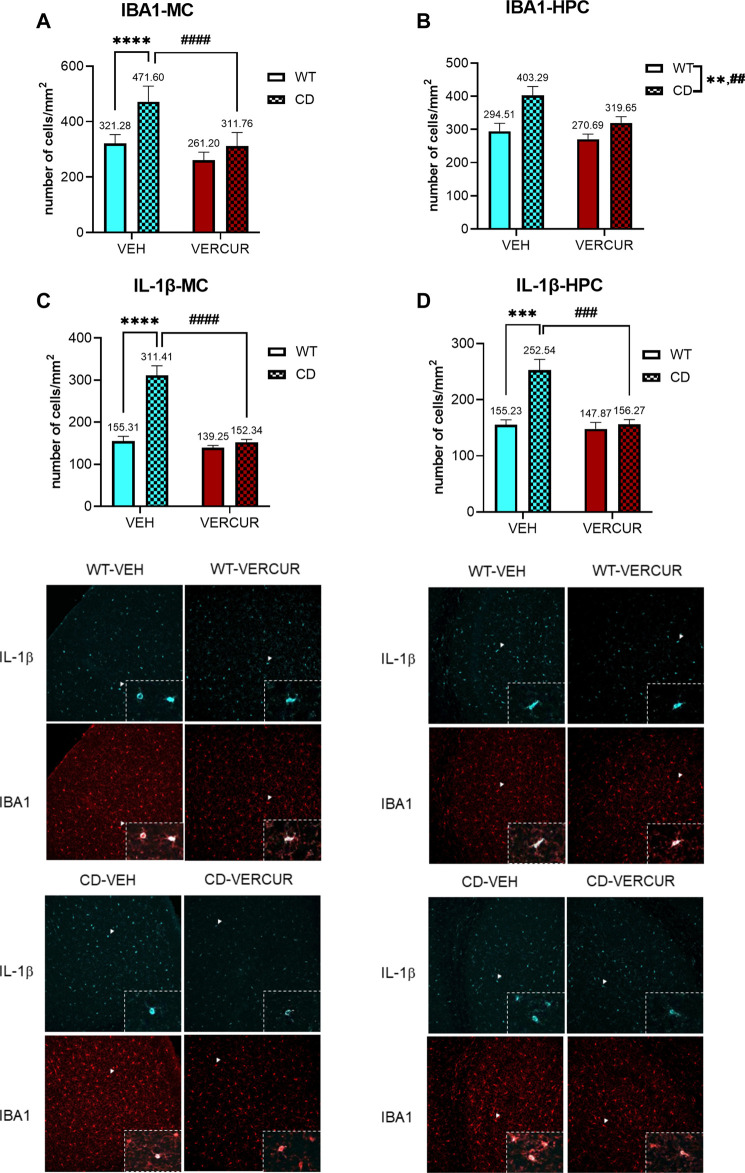
The increased microglia activation in the motor cortex and hippocampus presented by CD mice is prevented by VERCUR co-treatment. Quantification of IBA1 immunostaining in the MC **(A)** and HPC **(B)** and IL-1β immunostaining in the MC **(C)** and HPC **(D)** from VEH- and VERCUR-treated WT and CD animals. The panels on the bottom show representative images of IL-1β immunostaining (cyan) and IBA1 immunostaining (red). White arrowheads represent co-expression of IBA1 with IL-1β (zoomed in on the inset). The zoomed inset of the IBA1 image shows the cells co-expressing both markers (overlay). Images were taken at ×20. Data are presented as the mean ± SEM of cells per mm^2^ of n = 7–8 mice. *p* values are shown with asterisks (effect of genotype) and hashes (effect of treatment) indicating values that are significantly different in individual group comparisons (two-way ANOVA followed by Bonferroni’s *post hoc* test. ^**,##^ *p* < 0.01; ^***,###^ *p* < 0.001; ^****,####^ *p* < 0.0001.

Due to the fact that, in the brain of postmortem WBS patients, an increase in glia density has been described (Wilder et al., 2018), we wondered if the same could happen in our model and if the effective treatment in the improvement of some phenotypes could be related to this fact. Changes in microglial expression were evaluated in the MC and HPC of WT and CD mice after treatment with VEH or VERCUR. In the MC, statistical analysis revealed a significant interaction between genotype and treatment (F_1,25_ = 9.438, *p* = 0.0051). Subsequent post hoc analysis revealed a significant increase in IBA1 immunostaining in CD mice treated with VEH in comparison with the WT group (*p* < 0.0001), and VERCUR prevented this effect (*p* > 0.9999) (Figure 5A). A significant main effect of genotype (F_1,25_ = 12.90, *p* = 0.0014) and treatment (F_1,25_ = 5.988, *p* = 0.0218) but not interaction (F_1,25_ = 1.856, *p* = 0.1853) was also revealed in the HPC (Figure 5B).

In order to assess whether the activated microglia corresponded to the state M1 or M2, we analyzed IL-1β, as a pro-inflammatory cytokine, and TGF-β, as an anti-inflammatory cytokine. We evaluated the co-expression of IBA1 with IL-1β in WT and CD VEH- or VERCUR-treated mice. In the MC, the statistical analysis of IL-1β revealed a significant main effect of genotype, treatment, and interaction between factors (F_1,25_ = 24.56, *p* < 0.0001). Subsequent post hoc analysis revealed a significant increase in IL-1β in CD mice treated with VEH in comparison with the WT group (*p* < 0.0001), and VERCUR prevented this pro-inflammatory expression (*p* > 0.9999) (Figure 5C). Similarly, the expression of IL-1β in the HPC revealed a significant increase in IL-1β in CD mice treated with VEH in comparison with the WT group (*p* = 0.0001), and this was prevented after VERCUR co-treatment (*p* > 0.9999) (Figure 5D). Regarding TGF-β expression, no statistical differences were observed in the cortex or HPC of VEH-treated CD mice in comparison with the WT group (Supplementary Figure 6). See Supplementary Table 5 for statistical values.

### VERCUR Co-Treatment Prevents Gene Expression Changes in Cortex of CD Animals

To identify the molecular causes that could be related to the improvement in certain aspects of the behavioral phenotype in VERCUR-treated animals, we investigated differentially expressed genes (DEGs) by performing an RNA-seq analysis of the cerebral cortex from VERCUR-treated CD mice *versus* VEH-treated CD and WT mice.

**FIGURE 6:**
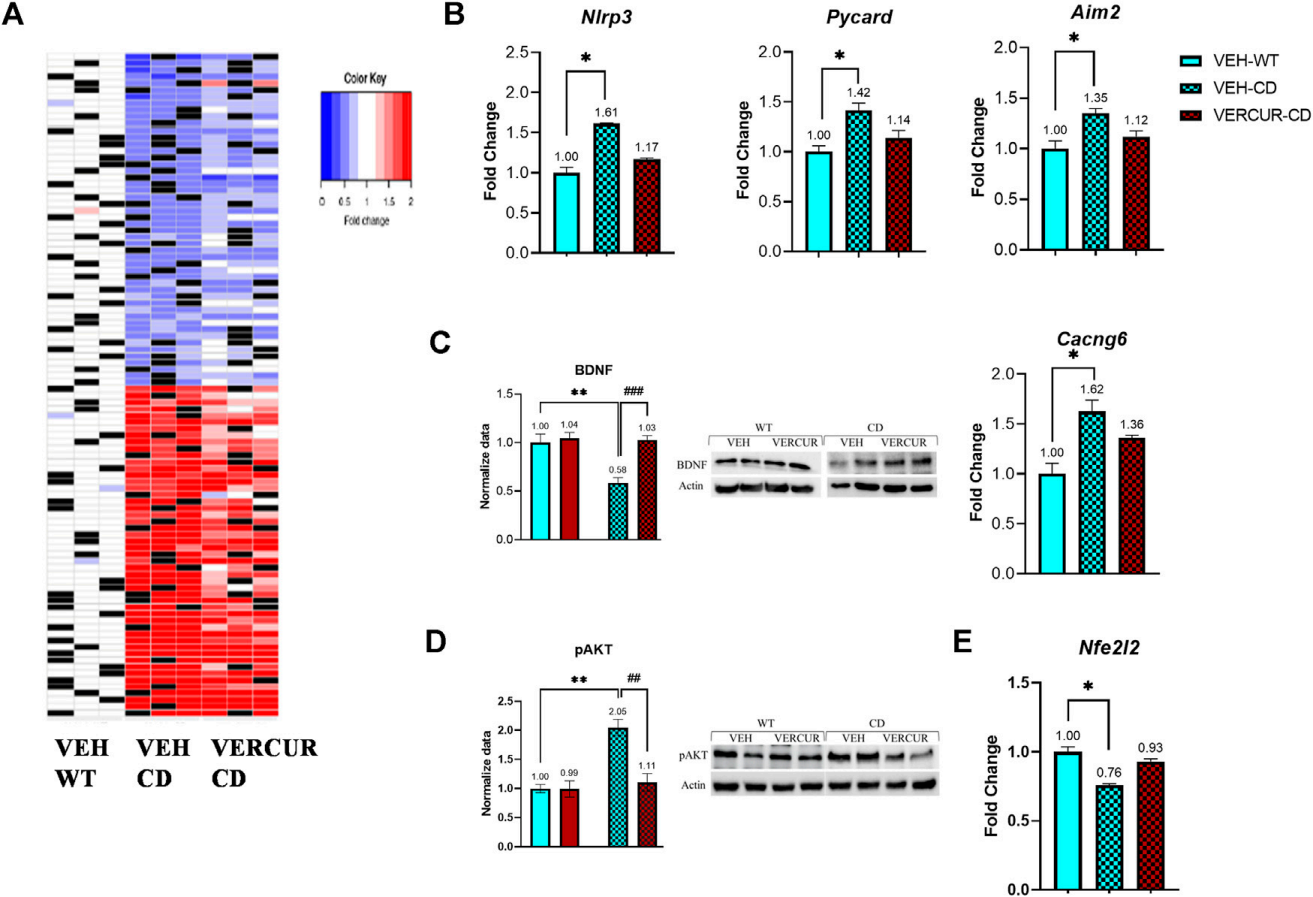
VERCUR co-treatment prevents gene expression changes in the cortex of CD animals. **(A)** Heatmap of the top 100 differentially expressed genes in the cortex. Top 50 up- and downregulated genes with the exclusion of genes included in the WBSCR. Black: no sample. Effects of VERCUR co-treatment on the gene expression levels of components of **(B)** the inflammosome pathway (*Nlrp3*, *Pycard*, *Aim2*) and **(C)** the MAPK signaling pathway (BDNF, *Cacng6*). **(D)** Quantification of pAKT levels. **(E)** VERCUR co-treatment restores the endogenous antioxidant *Nfe2l2* expression level. Data are presented as mean ± SEM. *p* values of multiple comparisons are shown with asterisks (effect of genotype) and hashes (effect of treatment) indicating values that are significantly different in individual group comparisons (Kruskal–Wallis test followed by Dunn’s *post hoc* test for RNA expression and two-way ANOVA followed by Bonferroni’s *post hoc* test for western blots, n = 4–7, with representative images on the right). ^*^ *p* < 0.05; ^**,##^ *p* < 0.01; ^###^ *p* < 0.001.

First, we conducted a targeted analysis of the genes in the WBSCR locus. Of the 26 genes that make up the WBSCR, only 19 were measurably expressed in the adult mouse cortex. As expected, all genes in the WBSCR region showed a decrease in the RNA amount in CD animals. We found 737 genes to be deregulated. The magnitude of these changes was generally small, with a fold-change ranking from 0.35 to 4.89. Differential expression analysis of VEH-treated CD and WT samples identified 424 genes with significantly increased expression and 313 genes with a decreased one. The expression of the top 100 DEGs clearly identified the two genotypes (Figure 6A) (Supplementary Table 6). When ordered by statistical significance, up- and downregulated genes had similar *p* values.

In order to investigate functional associations of the DEG, we performed a gene set enrichment analysis (GSEA) using the CPDB tool to identify significantly differentiated KEGG/Reactome pathways. The enrichment analysis performed between VEH-treated mice showed 18 pathways that were significantly altered (*q* ≤ 0.05) in CD compared to WT mice (Supplementary Table 7). Among them, the highest enrichment was observed in pathways related to inflammasomes (*q* < 0.0001), followed by the MAPK signaling pathway (*q =* 0.003), the NF-kappa B signaling pathway (*q =* 0.0088), G protein–coupled receptor (GPCR) ligand binding (*q =* 0.01), and extracellular matrix organization (*q =* 0.01) (Supplementary Table 8).

Next, we analyzed among the DEGs between VEH-treated mice those whose expression in VERCUR-treated CD mice was not significantly different from that in the VEH-treated WT mice. 53.6% of the genes recovered normal expression levels after co-treatment. Results of the GSEA with these genes showed eight differentiated pathways consistent with treatment rescue, including inflammasomes (*q* ≤ 0.001), extracellular matrix organization (*q* = 0.024), and MAPK signaling pathway (*q* = 0.046) (Table 1). In Table 2, we observe how co-treatment specifically affects each of the deregulated genes associated with the different pathways. Specifically, related to the activation of the inflammasome, we can see changes in genes such as *Nlrp3* (NLR family pyrin domain containing 3), *Pycard* (often referred to as ASC, apoptosis-associated speck-like protein containing a CARD), or *Aim2* (absent in melanoma 2) (Figure 6B). Related to the MAPK signaling pathway, we can observe changes in the expression of *Cacng6* (calcium voltage-gated channel auxiliary subunit gamma 6) and at protein levels in BDNF (Figure 6C). Furthermore, we observed that the increase in the amount of pAKT present in VEH-treated CD mice (*p* = 0.005) was also normalized in VERCUR-treated CD mice (Figure 6D). We also wanted to analyze M1/M2 related markers in detail. We can observe that M1 markers such as *Nos2*, *Il18*, or *Socs3* that are upregulated in VEH-treated CD mice normalize their expression after co-treatment, while others (*Cd68*, *Cd80*, and *Tlr2*) continue to appear upregulated (Supplementary Table 8). Expression changes are not observed in most of the M2 markers. Only two markers appear downregulated in VEH-treated CD mice (*Mrc1* and *Klf4*). We observed that co-treatment restores *Mrc1* levels, while it does not influence the expression of *Klf4* (Supplementary Table 8). In addition, we investigated the status of *Nfe2l2* (NRF2 coding gene) because there is growing evidence of a crosstalk between NRF2, MAPK, and inflammasomes. We found significantly reduced expression levels of this gene in VEH-treated CD mice that is normalized after VERCUR co-treatment (Figure 6E). See Supplementary Table 9 for statistical values.

**TABLE 1:** Pathway involvement of the differentially expressed genes recovered after treatment.

Pathway	Source	*p* value	*q* value	Included genes
Inflammasomes	Reactome	7.15.10^–8^	9.2310^–6^	*Nlrp3*; *Aim2*; *Txnip*; *Pycard*; *Pstpip1*; *Bcl2*
NOD-like receptor signaling pathway	KEGG	0.000386	0.012476	*Nlrp3*; *Nfkbia*; *Gsdmd*; *Aim2*; *Card9*; *Txnip*; *Pycard*; *Pstpip1*; *Nlrx1*; *Bcl2*
Extracellular matrix organization	Reactome	0.000952	0.024582	*Dcn*; *Ctsk*; *Itgb7*; *Tnxb*; *Capn12*; *Tmprss6*; *Mmp25*; *Adam12*; *Col28a1*; *Col6a1*; *Col16a1*; *Col2a1*; *Adamts14*
Striated muscle contraction	Reactome	0.002304	0.046613	*Tnnt1*; *Des*; *Tpm2*; *Myl1*
MAPK signaling pathway	KEGG	0.002970	0.046613	*Rac2*; *Rasgrp4*; *Fgf5*; *Fas*; *Gadd45b*; *Epha2*; *Dusp5*; *Dusp4*; *Bdnf*; *Dusp1*; *Flt3*; *Cacng6*
NF-kappa B signaling pathway	KEGG	0.004058	0.046613	*Lbp*; *Gadd45b*; *Nfkbia*; *Pidd1*; *Zap70*; *Bcl2*
Peptide ligand–binding receptors	Reactome	0.004171	0.046613	*Trh*; *Bdkrb2*; *Cxcr4*; *Grp*; *Qrfpr*; *Rxfp2*; *Npffr1*; *Pomc*; *Agtr2*
RAF/MAP kinase cascade	Reactome	0.004459	0.046613	*Rasgrp4*; *Il2rg*; *Spred2*; *Dusp5*; *Dusp4*; *Vwf*; *Dusp1*; *Fgf5*; *Artn*

**TABLE 2:** Normalized expression values of deregulated genes in the significantly altered pathways. Values are normalized with respect to the VEH-treated WT group. Those values recovered after treatment with VERCUR are indicated in bold.

Pathway	Gene	Mean VEH-treated CD	Mean VERCUR-treated CD	% Genes UP
Inflammasome (Reactome)	*Aim2*	1.351	**1.121**	71.42
	*Bcl2*	0.735	0.799	
	*Nlrc4*	1.472	1.389	
	*Nlrp3*	1.548	**1.154**	
	*Pstpip1*	1.403	**1.055**	
	*Pycard*	1.415	**1.138**	
	*Txnip*	0.742	**0.981**	
MAPK signaling pathway and PI3K–AKT signaling pathway (KEGG)	*Bcl2*	0.7356	0.799	38.2
	*Bdnf*	0.669	**0.957**	
	*Cacng6*	1.585	1.390	
	*Col2a1*	1.252	**0.883**	
	*Col6a1*	1.306	**1.108**	
	*Creb3l3*	2.611	1.298	
	*Ddit4*	0.676	0.749	
	*Dusp1*	0.628	**0.839**	
	*Dusp4*	0.490	**0.927**	
	*Dusp5*	0.529	**0.881**	
	*Dusp9*	1.820	1.463	
	*Eif4ebp1*	1.496	1.404	
	*Epha2*	0.621	**0.935**	
	*Fas*	0.692	**0.887**	
	*Fgf5*	0.720	**0.796**	
	*Flt3*	0.744	**0.875**	
	*Gadd45a*	0.509	0.525	
	*Gadd45b*	0.632	0.782	
	*Gng8*	0.696	0.762	
	*Hspa1l*	1.385	1.850	
	*Hspb1*	0.537	0.474	
	*Il1r1*	0.689	0.793	
	*Il2rg*	1.354	0.797	
	*Itgb7*	1.692	1.314	
	*Nr4a1*	0.3749	0.612	
	*Ntf3*	1.989	2.065	
	*Ppp2r1b*	0.654	0.730	
	*Rac2*	1.310	**1.042**	
	*Rasgrp4*	1.561	1.203	
	*Relb*	0.737	0.768	
	*Sgk1*	0.465	0.471	
	*Tnxb*	0.787	1.084	
	*Vegfd*	1.431	1.211	
	*Vwf*	0.847	0.890	
NK-kB (KEGG)	*Bcl2*	0.735	0.799	50
	*Card14*	1.860	1.887	
	*Gadd45b*	0.632	0.782	
	*Il1r1*	0.689	0.766	
	*Lbp*	1.312	**1.194**	
	*Lck*	1.663	1.467	
	*Nfkbia*	0.711	0.743	
	*Pidd1*	1.308	**1.185**	
	*Relb*	0.737	0.768	
	*Zap70*	1.311	**1.035**	
GPCR ligand binding (Reactome)	*Adm*	0.645	**1.019**	48.1
	*Adrb3*	1.485	1.337	
	*Agtr2*	0.614	0.434	
	*Bdkrb2*	1.376	1.284	
	*Ccr1*	4.891	2.985	
	*Cxcr4*	0.702	**1.004**	
	*Cysltr1*	0.670	**0.837**	
	*Fzd4*	0.712	**1.010**	
	*Fzd5*	0.677	**0.813**	
	*Glp2r*	1.816	1.559	
	*Gng8*	0.696	0.762	
	*Grp*	1.305	**1.100**	
	*Grpr*	0.553	**1.157**	
	*Npffr1*	1.376	1.272	
	*Nts*	0.686	0.484	
	*Opn3*	1.264	**1.169**	
	*Oprd1*	0.724	**0.885**	
	*Qrfpr*	0.704	0.716	
	*P2ry1*	0.698	**0.850**	
	*Pomc*	1.366	**0.914**	
	*Ptgdr*	1.614	1.776	
	*Pthlh*	0.623	**0.932**	
	*Rxfp2*	0.715	**0.829**	
	*Tacr3*	1.515	1.242	
	*Tbxa2r*	1.930	1.664	
	*Trh*	1.663	1.394	
	*Trpc6*	1.479	**1.134**	
Extracellular matrix organization (Reactome)	*Adam12*	0.699	0.763	73.7
	*Adamts14*	1.461	**1.221**	
	*Adam19*	0.671	**0.815**	
	*Capn12*	1.369	**0.915**	
	*Col16a1*	1.313	**1.158**	
	*Col17a1*	1.617	1.495	
	*Col2a1*	1.252	**0.883**	
	*Col28a1*	1.362	**0.950**	
	*Col6a1*	1.306	**1.108**	
	*Col7a1*	1.441	1.293	
	*Ctsk*	1.326	**1.173**	
	*Dcn*	0.728	**0.992**	
	*Gdf5*	2.126	1.797	
	*Itgb7*	1.692	1.314	
	*Mmp19*	2.031	1.548	
	*Mmp25*	0.594	**0.865**	
	*Optc*	1.757	**1.152**	
	*Tmprss6*	1.621	1.597	
	*Tnxb*	0.698	**1.115**	
NOD-like receptor signaling pathway (KEGG)	*Aim2*	1.351	**1.121**	75
	*Bcl2*	0.735	0.799	
	*Card9*	1.321	**0.871**	
	*Gsdmd*	1.281	1.231	
	*Nfkbia*	0.711	0.743	
	*Nlrc4*	1.472	1.389	
	*Nlrp3*	1.548	**1.154**	
	*Nlrx1*	1.250	**1.175**	
	*Oas2*	1.451	2.067	
	*Pstpip1*	1.403	**1.055**	
	*Pycard*	1.415	**1.138**	
	*Txnip*	0.742	**0.981**	

## Discussion

In this work, we analyzed the effect of a treatment combining curcumin, the most abundant phenol in turmeric, and verapamil, a widely used medication, on the CD murine model of WBS. The neurobehavioral phenotype of CD mice recapitulates most of the cognitive features observed in WBS patients, such as motor discoordination, hypersociability, and anxiety-like/compulsive behavior (Segura-Puimedon et al., 2014).

A significant improvement after co-treatment was observed in motor discoordination, evaluated with the rotarod test. Due to its effect on the modulation of calcium channels, verapamil has been studied as a therapeutic approach for different motor impairments such as dystonia or alcohol-induced motor discoordination. However, it has not given positive results in any of the cases (Jinnah et al., 2000; Baliño et al., 2010). In the case of curcumin, results have been contradictory. On the one hand, it has been described to significantly improve gait impairments in a model of α-synuclein deficit (Spinelli et al., 2015). On the other hand, it has been associated with detrimental effects on motor coordination in Huntington’s disease murine model, without affecting locomotor function or muscle strength (Hickey et al., 2012). In our study, none of the individual treatments had any effect, while the combinatorial treatment prevented motor discoordination in CD animals. This result reinforces the idea that the mechanisms of action of both molecules are different and complementarity is needed to obtain positive effects on the CD phenotype.

A significant positive effect of co-treatment on the social behavior of CD animals is also observed. VERCUR-treated CD mice showed a significantly better preference score than VEH-treated mice. Previous studies have defined that curcumin causes an increase in the exploratory behavior of stressed rats in an open field test (Vasileva et al., 2018). Our results on the evaluation of exploration time in sociability were not in agreement with this finding. Although the total exploration time seems to be increased in the group of WT animals treated with curcumin, these differences do not reach significant values.

Anxiety is one of the most frequent psychiatric disorders among WBS patients and present in more than 70% of patients, but it has some particularities (Dykens, 2003; Leyfer et al., 2006; Ng et al., 2016; Ng-Cordell et al., 2018). Using the elevated plus maze test, verapamil has shown anxiolytic effects in mouse and rat models (Gopala Krishna et al., 2001; Biala and Budzynska, 2006). In the same test, the use of curcumin for the treatment of anxiety-related behaviors has obtained contradictory results (Gilhotra and Dhingra, 2010; Kulkarni et al., 2011). In the previous analysis, a strong altered phenotype has been described in CD mice in the MBT (Borralleras et al., 2015; Ortiz-Romero et al., 2018; Dasilva et al., 2020). Although the MBT may not model anxiety or compulsive behavior *per se*, it is a good tool as a screening test to identify drugs with therapeutic potential for the treatment of these disorders (Jimenez-Gomez et al., 2011; de Brouwer et al., 2019). We did not observe any significant difference in any treated CD mice in the performance of this test.

We next aimed at gaining deeper insights into the neuropathological substrates that underlie the behavioral alterations observed in CD mice. Interestingly, previous studies have observed an increased glia density in postmortem brains of WBS patients (Wilder et al., 2018).We assessed the activation levels of microglia in the MC and the HPC. Our data showed a consistent increase in microglia activation in the MC and the HPC of CD in comparison with WT mice. Since activated microglia in the brain is associated with either an M1 (pro-inflammatory) or an M2 (anti-inflammatory) state, we evaluated which one was specifically affecting each of these phenotypes. Our results revealed an increase in the co-expression of IBA1 and the pro-inflammatory cytokine IL-1β. Notably, VERCUR co-treatment prevented the enhancement of M1 pro-inflammatory microglia expressing IL-1β in the MC and HPC of CD mice. We could not appreciate differences between VEH-treated mice in the expression of the anti-inflammatory marker TFGβ1.

Therefore, we next investigated the possible alterations in the expression of genes related to the VERCUR co-treatment in the cortex of CD mice versus VEH-treated CD and WT mice. Our findings revealed major changes in the cortex of CD mice mostly related to the inflammasome and MAPK signaling systems. Thus, in genes such as *Nlrp3*, *Pycard*, *Aim2* (inflammasome) and *Bdnf*, *Rac2*, *Cacng6*, and pAKT (MAPK signaling), expression levels were significantly altered in the cortex of VEH-treated CD mice compared to WT mice, and they presented normalized expression after co-treatment. From the RNA-seq data, we specifically analyzed M1/M2-related genes. We observed that several M1-related genes (such as *Il18*, *Nos2*, *Socs3*) appear upregulated, while genes related to M2 show no changes (*Ccl17*, *Tgfβ1*, *Tgm2*) or appear downregulated (*Klf4* and *Mrc1*). The effect of co-treatment on all of them is variable. Finally, we also found decreased *Nfe2l2* gene expression in the cortex of CD mice treated with vehicle compared to WT controls. Remarkably, VERCUR co-treatment increased *Nfe2l2* expression specifically in the cortex of CD mice.

A novel treatment targeting MAPK-related pathways has been described to improve motor function in a mouse model of Rett syndrome (Adams et al., 2020), and a BDNF increase in the motor-related cortex has been associated with improvements in motor coordination in the mouse (Krishnan and Nestler, 2008; Inoue et al., 2018). Thus, we suggest that the downregulation of *Bdnf* and its relation with MAPK signaling pathways may at least be partly responsible for the neurocognitive phenotype of CD animals, which is consistent with what has been described in previous studies (Segura-Puimedon et al., 2013; Borralleras et al., 2015; Ortiz-Romero et al., 2018). The positive effect of VERCUR co-treatment could be attributed to a combination of the different properties of each molecule. Curcumin has been described to induce *Bdnf* expression (Zhang et al., 2012; Nam et al., 2014), thus suggesting that this molecule is mainly responsible for the *Bdnf* increased expression in treated CD animals. However, previous studies in our group had determined that solely the increase of *Bdnf* expression is not enough to improve the cognitive features of these animals (Ortiz-Romero et al., 2018). It has been described that BDNF action is highly related to a tightly controlled ionic homeostasis (Li et al., 1999; Numakawa et al., 2001; Adasme et al., 2011). Ionic signaling, especially regarding calcium, is closely related to MAPK signaling pathways as they include many calcium-dependent proteins. Although more studies are needed on this topic, the results of this work suggest that verapamil is also modifying these affected pathways by contributing to the regulation of ionic homeostasis, which is crucial for the proper action of BDNF.

Additionally, in agreement with previous reports that correlate curcumin with the increase of *Nfe2l2* expression and nuclear translocation (Tapia et al., 2012; Fattori et al., 2015), *Nfe2l2* expression levels are normalized in VERCUR-treated CD mice. Since ROS was shown to regulate the activation of the NLRP3 inflammasome (Tschopp and Schroder, 2010), a high number of reports have demonstrated anti-inflammatory effects of NRF2-activating substances (including curcumin (Xu et al., 2018)) in different disease models associated with NLRP3 inflammasome inhibition (Hennig et al., 2018). Our results show that, after treatment with VERCUR, there is normalization in the expression levels of *Nlrp3* and *Aim2*, which could be due to the increase in the expression of *Nfe2l2*. However, *Nlcr4* levels do not change after treatment, which would be in line with previous studies showing that the NLRC4 inflammasome is not affected by *Nfe2l2* (Zhao et al., 2014).

GPCR ligand binding–related and extracellular matrix organization–related pathways were also found altered in cortical tissue of CD animals. GPCR activation is involved in a wide variety of physiological processes that include behavioral modulation through neurotransmitter-dependent activation, and deregulation of this process has been related with mood disorders and depression (Grammatopoulos, 2017). Conservation of extracellular matrix structures in the central nervous system has been described as fundamental for processes such as cell migration, neural growth, synaptogenesis, and synaptic plasticity, among others (Song and Dityatev, 2018). Interestingly, routes regarding extracellular matrix function have already been described as disease-relevant pathways in transcriptomic analysis performed on human WBS cell lines (Henrichsen et al., 2011; Khattak et al., 2015). Co-treatment was able to recover the deficits found in genes related to the organization of the extracellular matrix. Unfortunately, in our analyses, this recovery at the molecular level is not reflected at the neuroanatomical level, since the treated animals are similar to the untreated ones.

Taking into account the limitations of the study, especially the RNA-seq results that need further validation, the results suggest that the behavioral improvements observed in CD animals after treatment could be due to its effect on the inflammasome-related and MAPK and PI3K/AKT signaling–related pathways. Altogether, the evaluation of gene expression in the cerebral cortex contributes to the unraveling of mechanisms involved in the WBS cognitive profile. Nonetheless, it has to be taken into account that the same deletion is present in all tissues of the organism. Thus, it has to be considered that the observed alterations may have consequences in other organs and systems besides the brain, as all of the affected pathways are ubiquitously expressed and have a high relevance in a wide variety of cell processes.

In conclusion, we suggest that the hemizygous loss of WBSCR in the cerebral cortex of CD mice has a direct effect on the neuroinflammatory state of the brain, as well as on the expression of some genes related to synaptic signaling or extracellular matrix structure, which are crucial for a proper neural function. This may at least be partly responsible for the behavioral phenotype observed in CD animals. A treatment combining verapamil and curcumin is able to address different molecular targets and rescue some of those pathways, being a promising therapeutic approach for the cognitive phenotype of WBS patients.

## Data Availability

The datasets presented in this study can be found in online repositories. The names of the repository/repositories and accession number(s) can be found in the article/Supplementary Material.
