# Supplementary material for: Co-Treatment With Verapamil and Curcumin Attenuates the Behavioral Alterations Observed in Williams–Beuren Syndrome Mice by Regulation of MAPK Pathway and Microglia Overexpression
Source: Front Pharmacol. 2021 Aug 3;12:670785. doi: 10.3389/fphar.2021.670785 (PMC8369570; doi:10.3389/fphar.2021.670785)
Supplement: Supplementary file 1 [file DataSheet1.PDF]

Supplementary Material

1 Supplementary Data

Supplementary Material should be uploaded separately on submission. Please include any supplementary data, figures and/or tables. All supplementary files are deposited to FigShare for permanent storage and receive a DOI.

Supplementary material is not typeset so please ensure that all information is clearly presented, the appropriate caption is included in the file and not in the manuscript, and that the style conforms to the rest of the article. To avoid discrepancies between the published article and the supplementary material, please do not add the title, author list, affiliations or correspondence in the supplementary files.

2 Supplementary Figures and Tables

2.1 Supplementary Figures

Supplementary Figure 1: Treatment intake and body weight

A. The amount of drink per cage was quantified and normalized to the number of animals per cage (2 to 4) and to the time between each change (48 to 60 hours). Consumption was not significantly different between groups ($F_{2.005,30.08}$ = 0.4796, p = 0.6242).

B. Daily intake was not significantly different among VEH-WT and the rest of the groups ($F_{3,41}$ = 0.9419, p = 0.4292). A significant effect of genotype is observed because CD mice drink less regardless of treatment (($F_{1,41}$ = 8.402, p = 0.006)

C. None of the treatments changed the reduced body weight presented by CD mice compared to WT mice (effect of genotype $F_{1,84}$ = 146.4, p<0.0001)

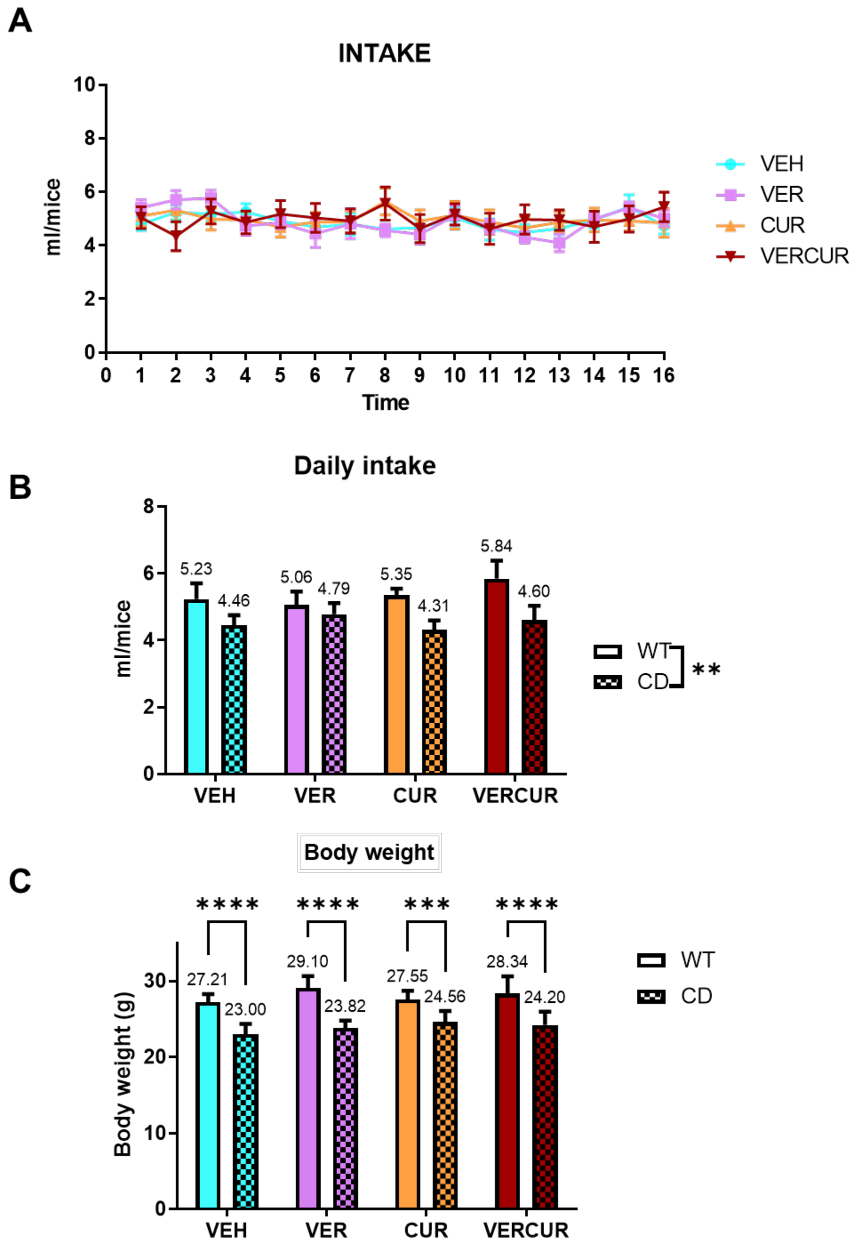

.

Supplementary Figure 2: WSCR genes expression analyses

Expression values were relativized according to the average expression of the WT animals for each gene. Expression analysis of the 20 genes included in the WS critical region (WSCR) represented in the RNAseq. VERCUR cotreatment don't change the expression levels of any of this genes. Data are presented as mean ± SEM of n=3 mice.

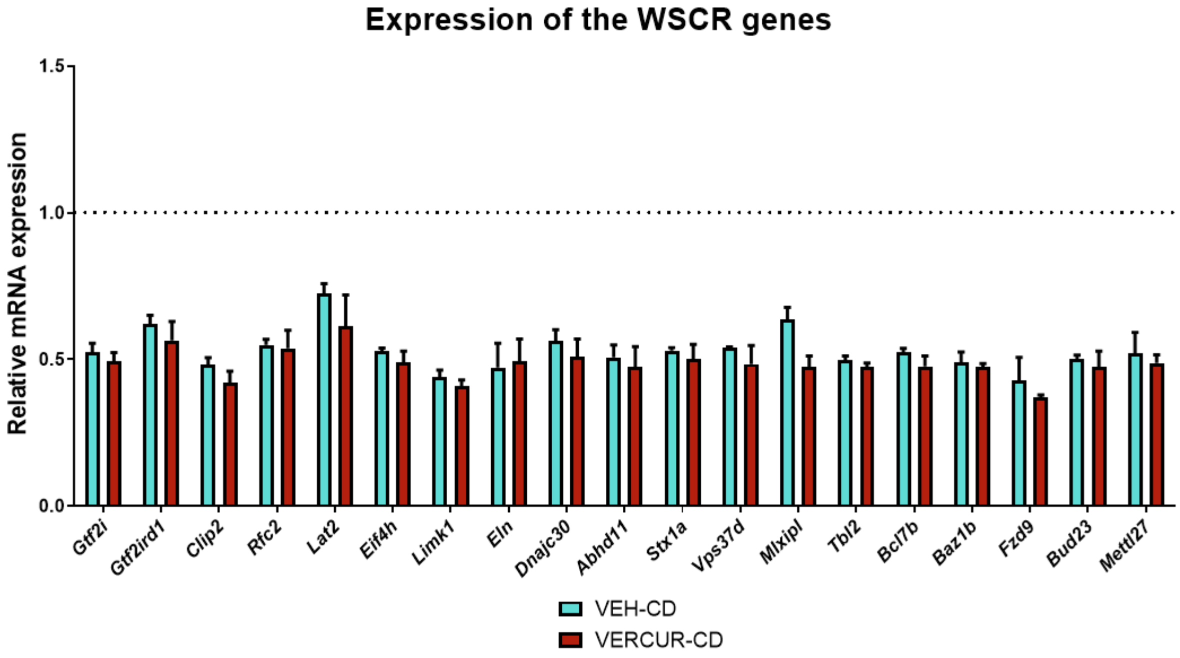

Supplementary Figure 3: Rotarod analysis of WT mice after treatment

None of the treatments had any effect in the performance of WT mice in this test. Data are presented as mean±SEM of n=7-11 mice.

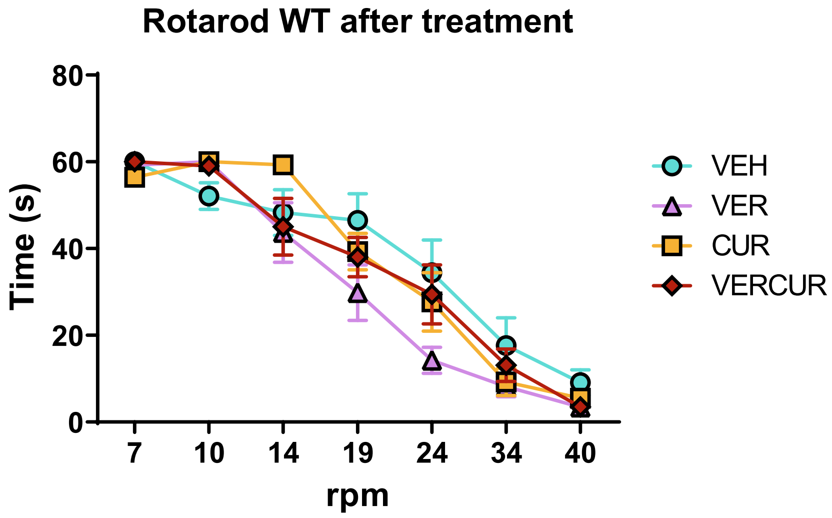

Supplementary Figure 4: Activity in the Sociability interaction test

No differences were observed, neither in genotype ($F_{1,69}$ = 1.235; p =0.2704) nor in treatment ($F_{3,69}$ = 1.166; p =0.3291) in the activity of the animals during the social interaction test. Data are presented as mean±SEM of n=8-11 mice.

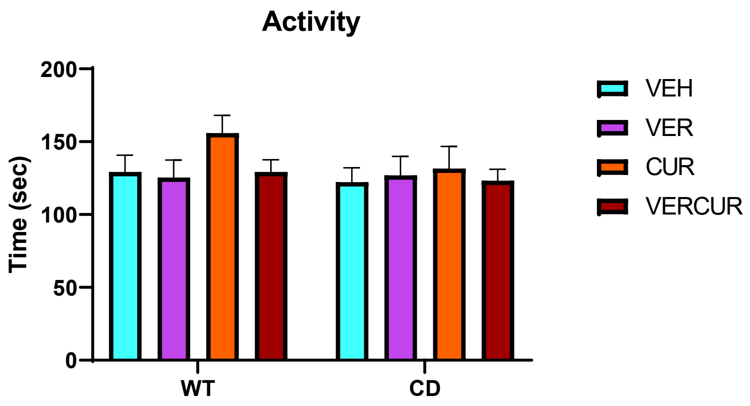

Supplementary Figure 5: Neuroanatomical Alterations in CD mice

A: We observed that none of the treatments had any effect in the recovery of a normal brain weight with a significant effect of genotype ($F_{1,67}$=171.2,p<0.0001) but no effect of treatment ($F_{3,67}$=0.2753,p=0.8431).

CD animals presented a significant reduction of the number of YFP+ neurons in both the MC **(B)** (effect of genotype: $F_{1,25}$=168.5, p<0.0001) and the HPC **(C)** (effect of genotype $F_{1,25}$=218.8, p<0.0001). None of the treatments had any effect on the recovery of the number of YFP+ neurons, neither in the MC ($F_{3,25}$=2.681, p=0.0686) nor in the HPC ($F_{3,25}$=1.427, p=0.2586)

Data are presented as mean ± SEM of n=8-11 (brain weight), n=3-5 mice (YFP quantification). *P* values are shown with asterisks indicating values that are significantly different in two-way ANOVA $^{****}p$<0.0001.

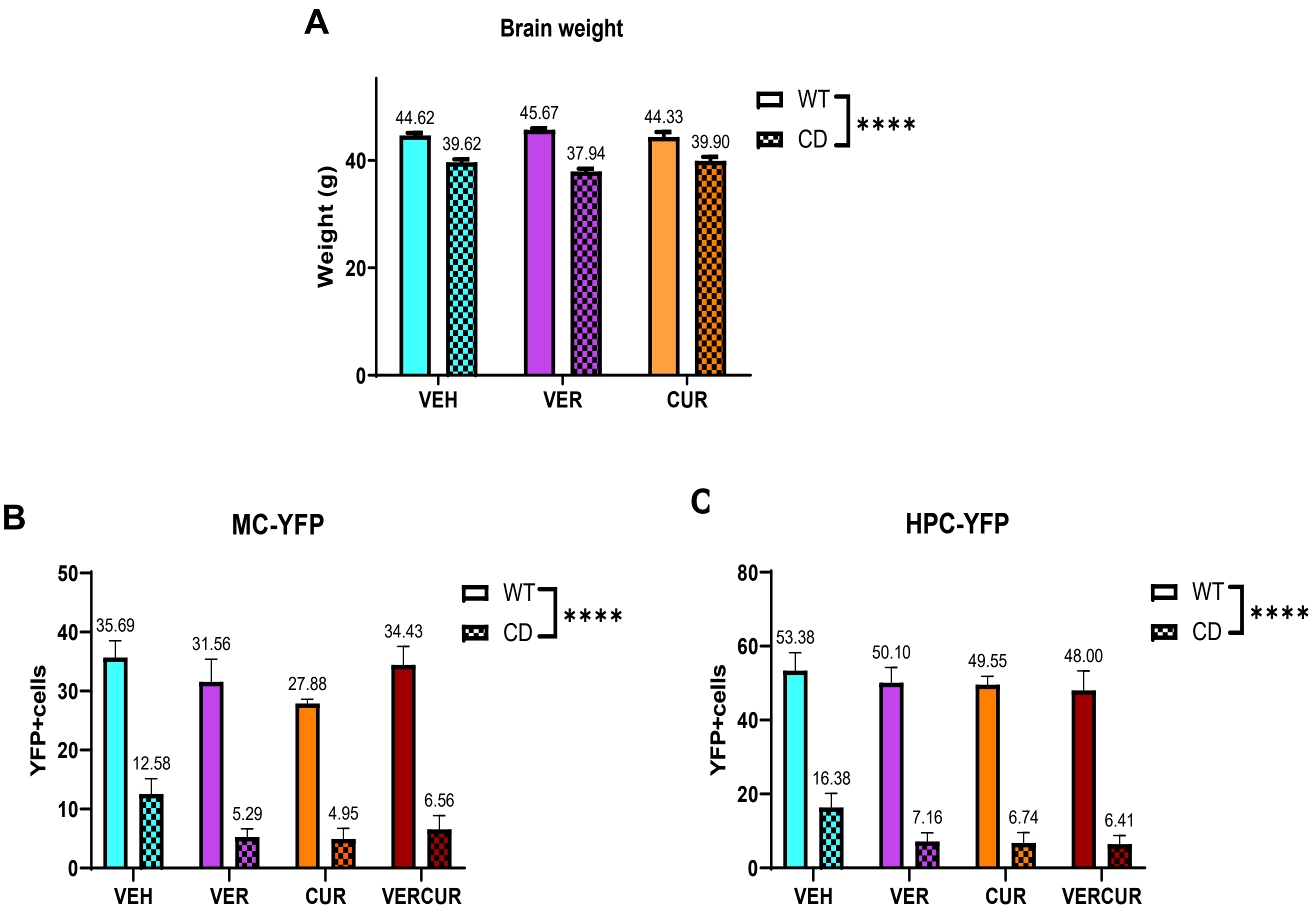

Supplementary Figure 6: TGFβ expression in VEH-treated mice

Quantification of TGFβ in the cortex (**A**) and in hippocampus (**B**) of VEH-treated WT and CD mice. There are no significant differences in any of the regions analyzed (Mann Whitney test; cortex: $p = 0.6095$; Hippocampus: $p = 0.8571$). Data are presented as mean ± SEM of n=4-6.

In the right representative western blot immunoblotted with anti- TGFβ. Bellow, ponceau staining for loading control.

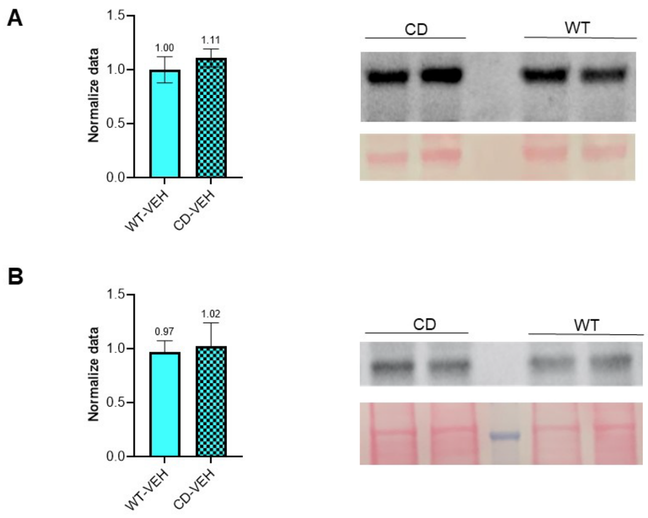

2.2 Supplementary Tables

Supplementary Table 1: Friedman Test and Kruskal-Wallis test with Dunn's multiple comparisons test of Motor Coordination

**Friedman test**	
P value	<0.0001
Number of groups	8
Friedman statistic	42.24
**Kruskal-Wallis test 7 rpm**	
P value	0.0001
Number of groups	8
Kruskal-Wallis statistic	29.14
**Dunn's multiple comparisons test**	
WT-VEH vs CD-VEH	0.0096
WT-VEH vs CD-VER	0.0104
WT-VEH vs CD-CUR	0.0446
WT-VEH vs CD-VERCUR	>0.9999

CD-VEH vs CD-VERCUR	0.0326
**Kruskal-Wallis test 10 rpm**	
P value	<0.0001
Number of groups	8
Kruskal-Wallis statistic	58.57
**Dunn's multiple comparisons test**	
WT-VEH vs CD-VEH	0.0018
WT-VEH vs CD-VER	0.0030
WT-VEH vs CD-CUR	0.0133
WT-VEH vs CD-VERCUR	>0.9999
CD-VEH vs CD-VERCUR	0.0032
**Kruskal-Wallis test 14 rpm**	
P value	<0.0001
Number of groups	8
Kruskal-Wallis statistic	49.11
**Dunn's multiple comparisons test**	
WT-VEH vs CD-VEH	0.0011
WT-VEH vs CD-VER	0.0003
WT-VEH vs CD-CUR	0.0050
WT-VEH vs CD-VERCUR	>0.9999
**Kruskal-Wallis test 19 rpm**	
P value	<0.0001
Number of groups	8
Kruskal-Wallis statistic	42.26
**Dunn's multiple comparisons test**	
WT-VEH vs CD-VEH	0.0002
WT-VEH vs CD-VER	<0.0001
WT-VEH vs CD-CUR	0.0010
WT-VEH vs CD-VERCUR	0.1714
**Kruskal-Wallis test 24 rpm**	
P value	<0.0001
Number of groups	8
Kruskal-Wallis statistic	31.44
**Dunn's multiple comparisons test**	
WT-VEH vs CD-VEH	0.0153
WT-VEH vs CD-VER	<0.0001
WT-VEH vs CD-CUR	0.0077
WT-VEH vs CD-VERCUR	0.1874
**Kruskal-Wallis test 34 rpm**	
P value	<0.0001
Number of groups	8
Kruskal-Wallis statistic	34.98
**Dunn's multiple comparisons test**	
WT-VEH vs CD-VEH	<0.0001

WT-VEH vs CD-VER	0.0002
WT-VEH vs CD-CUR	0.0033
WT-VEH vs CD-VERCUR	0.2166
**Kruskal-Wallis test 40 rpm**	
P value	0.2919
Number of groups	8
Kruskal-Wallis statistic	8.484

Supplementary Table 2: Two-way ANOVA of Sociability Test

Preference Score

**Source of variation**	**F (DFn, DFd)**	**P value**
Interaction	F (3, 69) = 2.876	0.0423
Genotype	F (1, 69) = 41.09	<0.0001
Treatment	F (3, 69) = 2.970	0.0378

**Bonferroni's multiple comparisons**	**Adjusted P Value**
WT-VEH vs CD-VEH	0.0064
WT-VEH vs CD-VER	0.0133
WT-VEH vs CD-CUR	0.0211
WT-VEH vs CD-VERCUR	>0.9999
CD-VEH vs CD-VERCUR	0.0469

Activity

**Source of variation**	**F (DFn, DFd)**	**P value**
Interaction	F (3, 69) = 0.4913	0.7242
Genotype	F (1, 69) = 1.235	0.2704
Treatment	F (3, 69) = 1.166	0.3291

Supplementary Table 3: Two-way ANOVA of Marble Burying Test

Source of variation	F (DFn, DFd)	P value
Interaction	F (3, 70) = 0.7893	0.5039
Genotype	F (1, 70) = 197.9	<0.0001
Treatment	F (3, 70) = 4.240	0.0082

Bonferroni's multiple comparisons	Adjusted P Value
WT-VEH vs CD-VEH	<0.0001
WT-VEH vs CD-VER	<0.0001
WT-VEH vs CD-CUR	<0.0001
WT-VEH vs CD-VERCUR	<0.0001
WT-VEH vs WT-VER	>0.9999
WT-VEH vs WT-CUR	>0.9999
WT-VEH vs WT-VERCUR	>0.9999

Supplementary Table 4: Statistical analysis of Neuroanatomy

Two-way ANOVA of Brain weight

**Source of Variation**	**F (DFn, DFd)**	**P value**
Interaction	F (3, 67) = 2.468	0.0696
Genotype	F (1, 67) = 171.2	<0.0001
Treatment	F (3, 67) = 0.2753	0.8431

Two-way ANOVA of YFP+Cells-MC

**Source of Variation**	**F (DFn, DFd)**	**P value**
Interaction	F (3, 25) = 0.4021	0.7527
Genotype	F (1, 25) = 168.5	<0.0001
Treatment	F (3, 25) = 2.681	0.0686

Two-way ANOVA of YFP+Cells-HPC

**Source of Variation**	**F (DFn, DFd)**	**P value**
Interaction	F (3, 25) = 0.2273	0.8765
Genotype	F (1, 25) = 218.8	<0.0001
Treatment	F (3, 25) = 1.427	0.2586

Supplementary Table 5: Statistical analysis of Microglia

Two-way ANOVA of IBA 1 in MC

Source of Variation	F (DFn, DFd)	P value
Interaction	F (1, 25) = 9.438	0.0051
Treatment	F (1, 25) = 45.87	<0.0001
Genotype	F (1, 25) = 38.27	<0.0001
**Bonferroni's multiple comparisons test**	**Adjusted P Value**	
WT-VEH vs CD-VEH	<0.0001	
WT-VEH vs CD-VERCUR	>0.9999	
WT-VEH vs WT-VERCUR	0.0978	
CD-VEH vs CD-VERCUR	<0.0001	

Two-way ANOVA of IBA 1 in HPC

Source of Variation	F (DFn, DFd)	P value
Interaction	F (1, 25) = 1.856	0.1853
Treatment	F (1, 25) = 5.988	0.0218
Genotype	F (1, 25) = 12.90	0.0014

Two-way ANOVA of IL-1b in MC

Source of Variation	F (DFn, DFd)	P value
Interaction	F (1, 25) = 24.56	<0.0001
Treatment	F (1, 25) = 36.85	<0.0001
Genotype	F (1, 25) = 34.37	<0.0001
**Bonferroni's multiple comparisons test**	**Adjusted P Value**	
WT-VEH vs CD-VEH	<0.0001	
WT-VEH vs CD-VERCUR	>0.9999	
WT-VEH vs WT-VERCUR	>0.9999	
CD-VEH vs CD-VERCUR	<0.0001	

Two-way ANOVA of IL-1b in HPC

Source of Variation	F (DFn, DFd)	P value
Interaction	F (1, 25) = 2.887	0.0031
Treatment	F (1, 25) = 6.024	0.0008
Genotype	F (1, 25) = 5.496	0.0006
**Bonferroni's multiple comparisons test**	**Adjusted P Value**	
WT-VEH vs CD-VEH	0.0001	
WT-VEH vs CD-VERCUR	>0.9999	
WT-VEH vs WT-VERCUR	>0.9999	
CD-VEH vs CD-VERCUR	0.0002	

**Mann Whitney test of TGFβ in Cortex**	**U**	**P value**
**Cortex**	9	0.6095
**Hippocampus**	9	0.8571

Supplementary Table 6: List of the TOP 50 downregulated and Upregulated genes

**TOP 50 Downregulated**			
**ENSMUSG-ID**	**ID**	**Mean VEH-CD**	**Mean VERCUR-CD**
ENSMUSG00000039997.16	*Ifi203*	0,348983643	0,536204235
ENSMUSG00000024042.7	*Sik1*	0,386996917	0,670001663
ENSMUSG00000034732.4	*Pabpc5*	0,405282423	0,731954733
ENSMUSG00000074170.5	*Plekhf1*	0,41468441	0,452475973
ENSMUSG00000015702.13	*Anxa9*	0,41808629	1,421389519
ENSMUSG00000006764.8	*Tph2*	0,4275309	0,631133782
ENSMUSG00000034936.2	*Arl4d*	0,440062518	0,663663814
ENSMUSG00000034640.9	*Tiparp*	0,456783995	0,696754443
ENSMUSG00000032515.8	*Csrnp1*	0,459576168	0,726705454
ENSMUSG00000030787.4	*Lyve1*	0,461300882	0,631605128
ENSMUSG00000024427.6	*Spry4*	0,461340128	0,756686652
ENSMUSG00000019970.15	*Sgk1*	0,46529929	0,471987981
ENSMUSG00000005124.10	*Wisp1*	0,47265273	0,675172642
ENSMUSG00000023232.17	*Serinc2*	0,479939088	0,612273739
ENSMUSG00000040298.6	*Btbd16*	0,486687401	0,649812423
ENSMUSG00000029641.8	*Rasl11a*	0,487319758	0,634220111
ENSMUSG00000037465.10	*Klf10*	0,489450943	0,649050024
ENSMUSG00000031530.6	*Dusp4*	0,490155833	0,927443584
ENSMUSG00000094786.1	*Gm14403*	0,494628012	0,423999306
ENSMUSG00000022769.9	*Sdf2l1*	0,496297155	0,507753103
ENSMUSG00000078866.10	*Zfp970*	0,498476893	0,534981018
ENSMUSG00000028214.13	*Gem*	0,507226979	0,66659189
ENSMUSG00000015890.2	*Amdhd1*	0,511609576	0,666004972
ENSMUSG00000055148.7	*Klf2*	0,514962481	0,566931336
ENSMUSG00000040170.13	*Fmo2*	0,515795186	0,85292683
ENSMUSG00000037992.16	*Rara*	0,521018761	0,559406489
ENSMUSG00000073158.4	*9030624G23Rik*	0,526585295	0,55496269
ENSMUSG00000034765.6	*Dusp5*	0,529014939	0,88159771
ENSMUSG00000028680.14	*Plk3*	0,530060839	0,755284108
ENSMUSG00000051678.4	*Pcdhb6*	0,531541899	0,597623225
ENSMUSG00000004951.10	*Hspb1*	0,537998949	0,474504863
ENSMUSG00000022367.7	*Has2*	0,53915411	0,843441436
ENSMUSG00000021464.14	*Ror2*	0,539486693	0,993362668
ENSMUSG00000040812.14	*Agbl2*	0,541919585	0,681546383
ENSMUSG00000019848.14	*Popdc3*	0,554067944	0,506816815
ENSMUSG00000039457.4	*Ppl*	0,558201204	0,607014596
ENSMUSG00000047330.8	*Kcne4*	0,55822905	0,724447742

ENSMUSG00000041301.15	*Cftr*	0,56001384	0,793214998
ENSMUSG00000050919.9	*Zfp366*	0,560835325	0,811177096
ENSMUSG00000037482.2	*Erv3*	0,567555099	0,538114735
ENSMUSG00000039634.12	*Zfp189*	0,569922613	0,99319742
ENSMUSG00000037112.16	*Sik2*	0,57803539	0,480872311
ENSMUSG00000091405.2	*Hist2h4*	0,578884758	0,581370777
ENSMUSG00000109293.1	*Dcst2*	0,580347286	0,908775565
ENSMUSG00000039676.4	*Capsl*	0,581570979	0,787983212
ENSMUSG00000048534.7	*Jaml*	0,583471551	0,820073392
ENSMUSG00000061816.15	*Myl1*	0,583505887	0,823037669
ENSMUSG00000043832.13	*Clec4a3*	0,584104772	0,928522514
ENSMUSG00000069727.5	*Zfp975*	0,584234849	0,614783083
ENSMUSG00000035621.13	*Midn*	0,584654504	0,799069887
**TOP 50 Upregulated**			
ENSMUSG00000025804.5	*Ccr1*	4,891969222	2,985885629
ENSMUSG00000094777.2	*Hist1h2ap*	3,725802935	4,740589031
ENSMUSG00000001493.9	*Meox1*	3,344147255	2,248741179
ENSMUSG00000042102.7	*Dmgdh*	3,231422342	3,336435202
ENSMUSG00000024124.10	*Prss30*	2,98095855	3,533839072
ENSMUSG00000034762.9	*Glis1*	2,78954374	1,592887859
ENSMUSG00000035041,8	*Creb3l3*	2,611737805	1,494817073
ENSMUSG00000074210.3	*E130208F15Rik*	2,605543191	2,571718838
ENSMUSG00000094152.5	*Slc6a16*	2,602728939	2,048253534
ENSMUSG00000092124.1	*B930094E09Rik*	2,592499181	1,489039728
ENSMUSG00000041202,12	*Pla2g2d*	2,454332553	2,71090294
ENSMUSG00000042895.6	*Abra*	2,40436573	1,834576014
ENSMUSG00000037962.7	*Rflna*	2,365999878	2,001353403
ENSMUSG00000019368.13	*Sec14l4*	2,313978574	2,232229775
ENSMUSG00000090317.1	*Gm17324*	2,269498818	1,64294922
ENSMUSG00000032015.16	*Pou2f3*	2,238399006	2,149540122
ENSMUSG00000039098.3	*Gm9767*	2,192653433	1,390177301
ENSMUSG00000046610.14	*Oacyl*	2,182646431	1,749083176
ENSMUSG00000047257.13	*Prss45*	2,165913867	1,845481022
ENSMUSG00000000320.10	*Alox12*	2,144391689	1,62093523
ENSMUSG00000055235.11	*Wdr86*	2,132439568	2,354902523
ENSMUSG00000038259,4	*Gdf5*	2,12615494	1,797085999
ENSMUSG00000027456.8	*Sdcbp2*	2,113774048	1,648650878
ENSMUSG00000050621.7	*Rps27rt*	2,057248974	1,376070656
ENSMUSG00000079051.5	*Gm14025*	2,057182373	1,32407773
ENSMUSG00000040632.16	*Nrl*	2,050549734	1,275468174
ENSMUSG00000096140.2	*Ankrd66*	2,038567709	2,076095281
ENSMUSG00000028860.13	*Sytl1*	2,03390529	1,490862557
ENSMUSG00000025355,7	*Mmp19*	2,031675875	1,548066298
ENSMUSG00000032717.14	*Mdfi*	1,994846943	1,793076711

ENSMUSG00000049107.13	*Ntf3*	1,989050995	2,065151362
ENSMUSG00000071497.3	*Nutf2-ps1*	1,972574672	1,534807276
ENSMUSG00000066878.5	*Gm10184*	1,957507205	1,859719759
ENSMUSG00000034881.8	*Tbxa2r*	1,930958942	1,664141499
ENSMUSG00000044006.8	*Cilp2*	1,909356945	1,42166311
ENSMUSG00000053318.7	*Slamf8*	1,876998974	1,808240508
ENSMUSG00000046750.17	*Selenov*	1,872842035	1,414601698
ENSMUSG00000028011.16	*Tdo2*	1,863005171	0,901047593
ENSMUSG00000013483.14	*Card14*	1,8601762820	1,8878205128
ENSMUSG00000045915.15	*Ccdc42*	1,855891529	1,570499552
ENSMUSG00000046245.13	*Pilra*	1,854700781	1,910051684
ENSMUSG00000096753.7	*Fam181a*	1,853808488	1,876249925
ENSMUSG00000051224.13	*Tceanc*	1,8327315	1,643857188
ENSMUSG00000075122,5	*Cd80*	1,832406892	1,3110061145
ENSMUSG00000048070.4	*Pirt*	1,8261485	2,479752217
ENSMUSG00000031383.8	*Dusp9*	1,820406723	1,46364426
ENSMUSG00000024430.14	*Cabyr*	1,820095982	1,535612569
ENSMUSG00000049928.15	*Glp2r*	1,816858953	1,559896426
ENSMUSG00000035692.6	*Isg15*	1,810119909	1,154789323
ENSMUSG00000056162.13	*Cndp1*	1,808070451	1,263666461

Supplementary Table 7: Pathway involvement of the differentially expressed genes between vehicle-treated WT and CD mice

**Pathway**	**Source**	***p* value**	***q* value**	**Included genes**
Inflammasomes	Reactome	4,60E-08	1,09E-05	*Nlrp3; Aim2; Txnip; Nlrc4; Pycard; Pstpip1; Bcl2*
MAPK signaling pathway	KEGG	3,90E-05	0.00307	*Fas; Ntf3; Rac2; Rasgrp4; Hspb1; Fgf5; Gadd45a; Gadd45b; Nr4a1; Epha2; Vegfd; Dusp9; Il1r1; Relb; Dusp5; Dusp4; Bdnf; Dusp1; Flt3; Cacng6; Hspa1l*
NF-kappa B signaling pathway	KEGG	0.00018	0.00884	*Lbp; Gadd45b; Nfkbia; Pidd1; Zap70; Relb; Card14; Lck; Il1r1; Bcl2*
GPCR ligand binding	Reactome	0.00027	0.01072	*Glp2r; Tbxa2r; Ccr1; Ptgdr; Opn3; Cysltr1; Adm; Fzd4; Fzd5; Rxfp2; Pthlh; Cxcr4; Trh; Grp; Npffr1; Pomc; Grpr; P2ry1; Tacr3; Bdkrb2; Nts; Adrb3; Oprd1; Agtr2; Qrfpr; Gng8*
Extracellular matrix organization	Reactome	0.00031	0.01072	*Dcn; Ctsk; Adamts14; Col7a1; Itgb7; Tnxb; Capn12; Tmprss6; Mmp19; Mmp25; Adam12; Optc; Col28a1; Gdf5; Col6a1; Adam19; Col16a1; Col2a1; Col17a1*
Class A/1 (Rhodopsin-like receptors)	Reactome	0.00050	0.01505	*Trh; Grpr; Bdkrb2; Grp; Tbxa2r; Tacr3; Qrfpr; Rxfp2; Npffr1; Ccr1; Pomc; Adrb3; Oprd1; Cxcr4; Ptgdr; Agtr2; P2ry1; Opn3; Nts; Cysltr1*

Collagen chain trimerization	Reactome	0.00099	0.02359	*Col7a1; Col28a1; Col6a1; Col16a1; Col2a1; Col17a1*
PI3K-Akt signaling pathway	KEGG	0.00117	0.02523	*Bdnf; Ntf3; Ddit4; Itgb7; Sgk1; Creb3l3; Nr4a1; Tnxb; Ppp2r1b; Epha2; Vegfd; Il2rg; Eif4ebp1; Col6a1; Vwf; Fgf5; Col2a1; Flt3; Gng8; Bcl2*
Arachidonic acid metabolism	KEGG	0.00132	0.02624	*Fam213b; Pla2g2d; Gpx3; Pla2g3; Plb1; Hpgds; Alox12*
Pathways in cancer	KEGG	0.00168	0.02908	*Rasgrp4; Nfkbia; Nfe2l2; Flt3; Rara; Heyl; Fzd4; Zbtb16; Fas; Gadd45b; Cxcr4; Ctnna3; Dapk2; Rac2; Vegfd; Fgf5; Fzd5; Bdkrb2; Il2rg; Frat1; Stat5a; Nkx3-1; Gadd45a; Bcl2; Gng8; Hhip*
NOD-like receptor signaling pathway	KEGG	0.00171	0.02908	*Nlrp3; Nfkbia; Gsdmd; Aim2; Txnip; Card9; Oas2; Nlrc4; Pycard; Pstpip1; Nlrx1; Bcl2*
Degradation of the extracellular matrix	Reactome	0.00185	0.02934	*Dcn; Ctsk; Mmp19; Tmprss6; Capn12; Optc; Mmp25; Col16a1; Col17a1*
Collagen biosynthesis and modifying enzymes	Reactome	0.00208	0.02975	*Col7a1; Col28a1; Col6a1; Col16a1; Col17a1; Col2a1; Adamts14*
Striated Muscle Contraction	Reactome	0.00213	0.02975	*Tnnt1; Tpm2; Des; Tnnc1; Myl1*
RAF-independent MAPK1/3 activation	Reactome	0.00287	0.03580	*Dusp5; Dusp4; Dusp1; Dusp9*

Histidine metabolism	KEGG	0.00287	0.03580	*Amdhd1; Aldh3b1; Cndp1; Aldh3b2*
Negative regulation of MAPK pathway	Reactome	0.00388	0.04603	*Dusp5; Dusp4; Ppp2r1b; Dusp1; Dusp9*
G alpha (q) signaling events	Reactome	0.00445	0.05023	*Trh; Bdkrb2; Grp; Tbxa2r; Qrfpr; Npffr1; Trpc6; Grpr; P2ry1; Tacr3; Nts; Gng8; Cysltr1*

Supplementary Table 8: Expression data of M1/M2 related genes

	ENSMUSG-ID	ID	Mean VEH-CD	Mean VERCUR-CD
**M1 Markers**	ENSMUSG00000018774,13	*Cd68*	1,473528644	1,320664264
	ENSMUSG00000075122,5	*Cd80*	1,832406893	1,311006115
	ENSMUSG00000022901,13	*Cd86*	0,899077459	0,93305483
	ENSMUSG00000066026,14	*Dhrs3*	1,00557545	0,795328115
	ENSMUSG00000027776,12	*Il12a*	1,117674763	1,064354437
	ENSMUSG00000039217,13	*Il18*	1,295965825	1,122771174
	ENSMUSG00000026072,12	*Il1r1*	0,834421483	0,843618712
	ENSMUSG00000020826,8	*Nos2*	1,283880171	0,894695889
	ENSMUSG00000026177,11	*Slc11a1*	1,003997986	0,840347084
	ENSMUSG00000053113,3	*Socs3*	1,281579009	0,980460817
	ENSMUSG00000029304,14	*Spp1*	1,006009224	0,858396636
	ENSMUSG00000027995,10	*Tlr2*	1,437470726	1,245745511
	ENSMUSG00000039005,13	*Tlr4*	1,319939917	0,886844411
**M2 Markers**	ENSMUSG00000031780,2	*Ccl17*	1,046704722	0,772357724
	ENSMUSG00000008845,9	*Cd163*	1,053589041	1,216547945
	ENSMUSG00000022661,14	*Cd200*	0,985995354	1,056527989
	ENSMUSG00000022667,18	*Cd200r1*	0,862528612	0,950047125
	ENSMUSG00000003032,8	*Klf4*	0,690413251	0,692691784
	ENSMUSG00000026712,3	*Mrc1*	0,602075189	1,044269317
	ENSMUSG00000002603,15	*Tgfb1*	1,049371949	0,967055396
	ENSMUSG00000037820,15	*Tgm2*	0,948300618	0,83114715

Supplemental Table 9: Statistical analysis of Expression markers

Kruskal-Wallis test of *Nlrp3*

**H (ng)**	**P value**
H (3) = 7.200	0.0036
**Dunn's multiple comparisons test**	**Adjusted P Value**
WT-VEH vs. CD-VEH	0,0146
WT-VEH vs. CD-VERCUR	0,3594

Kruskal-Wallis test of *Pycard*

**H (ng)**	**P value**
H (3) = 6.489	0.0107
**Dunn's multiple comparisons test**	**Adjusted P Value**
WT-VEH vs. CD-VEH	0,0225
WT-VEH vs. CD-VERCUR	0,5934

Kruskal-Wallis test of *Aim2*

**H (ng)**	**P value**
H (3) = 6.489	0.0107
**Dunn's multiple comparisons test**	**Adjusted P Value**
WT-VEH vs. CD-VEH	0,0225
WT-VEH vs. CD-VERCUR	0,5934

Kruskal-Wallis test of *Cacng6*

**H (ng)**	**P value**
H (3) = 7.200	0.0036
**Dunn's multiple comparisons test**	**Adjusted P Value**
WT-VEH vs. CD-VEH	0,0146
WT-VEH vs. CD-VERCUR	0,3594

Kruskal-Wallis test of *Nfe2l2*

**H (ng)**	**P value**
H (3) = 6.489	0.0107
**Dunn's multiple comparisons test**	**Adjusted P Value**
WT-VEH vs. CD-VEH	0,0225
WT-VEH vs. CD-VERCUR	0,5934

Two-way ANOVA of BDNF

**Source of Variation**	**F (DFn, DFd)**	**P value**
Interaction	F (1, 14) = 10.34	0.0062
Treatment	F (1, 14) = 15.24	0.0016
Genotype	F (1, 14) = 11.92	0.0039
**Bonferroni's multiple comparisons test**	**Adjusted P Value**	
WT-VEH vs CD-VEH	0.0029	

WT-VEH vs CD-VERCUR	>0.9999
WT-VEH vs WT-VERCUR	>0.9999
CD-VEH vs CD-VERCUR	0.0007

Two-way ANOVA of pAKT

**Source of Variation**	**F (DFn, DFd)**	**P value**
Interaction	F (1, 14) = 11.04	0.0050
Treatment	F (1, 14) = 11.35	0.0046
Genotype	F (1, 14) = 17.07	0.0010
**Bonferroni's multiple comparisons test**	**Adjusted P Value**	
WT-VEH vs CD-VEH	0.0011	
WT-VEH vs CD-VERCUR	>0.9999	
WT-VEH vs WT-VERCUR	>0.9999	
CD-VEH vs CD-VERCUR	0.0012	
